# Resetting the epigenetic clock: cellular senescence and regenerative strategies in intervertebral disc degeneration

**DOI:** 10.3389/fragi.2026.1842955

**Published:** 2026-06-17

**Authors:** Minfan Li, Haitao Deng, Hongda Xu, Qingzhong Zhou, Shengxin Zhao

**Affiliations:** 1 Spinal Surgery Department, Mianyang Orthopaedic Hospital, Mianyang, China; 2 Department of Orthopedic Surgery, The Affiliated Hospital of Southwest Medical University, Luzhou, China

**Keywords:** cellular senescence, epigenetics, intervertebral disc degeneration, reprogramming, senescence-associated secretory phenotype

## Abstract

Intervertebral disc degeneration (IDD) is the leading pathological cause of low back pain, while current clinical treatments are only palliative and cannot reverse the programmed cellular senescence driven by epigenetic dysregulation. This process is characterized by progressive loss of nucleus pulposus (NP) cell identity and establishment of a self-amplifying senescence-associated microenvironment. In this review, we synthesize recent advances elucidating how heterogeneous senescent cell populations and their secretory phenotype (SASP) orchestrate a destructive vicious cycle in IDD. We further dissect the synergistic interplay among DNA methylation, histone modifications, and non-coding RNAs that constitutes the “epigenetic aging clock” and drives premature cellular aging within the disc. Notably, we evaluate emerging therapeutic strategies aimed at clock reversal, including senolytic clearance of senescent cells, epigenetic remodeling using small-molecule inhibitors or CRISPR-dCas9 editing, and cellular reprogramming approaches ranging from iPSC differentiation to direct lineage conversion. We propose a synergistic “clear, prime, then seed” roadmap that sequentially combines these interventions for optimal regeneration. This work provides a systematic theoretical framework for the clinical translation of epigenetic-targeted therapy for IDD, and breaks through the cognitive limitation of traditional mechanical wear theory.

## 1 Introduction

Low back pain is the leading cause of disability worldwide, with intervertebral disc degeneration (IDD) serving as its most prevalent pathological basis (GBD, 2021 US Burden of Disease Collaborators, 2024; Knezevic et al., 2021). The socioeconomic burden associated with IDD continues to escalate in parallel with the aging population (Dowdell et al., 2017). Current clinical interventions—ranging from conservative analgesia to surgical procedures—primarily aim to alleviate symptoms or restore mechanical stability. However, these approaches fail to reverse the underlying biological progression of degeneration and remain fundamentally palliative in nature (Risbud and Shapiro, 2014). This clinical impasse necessitates a fundamental re-evaluation of the pathogenesis of IDD.

Conventional perspectives have conceptualized IDD as a passive structural attrition driven by mechanical overloading, reduced nutrient supply, and metabolic dysregulation, ultimately culminating in the progressive degradation of the extracellular matrix (Vo et al., 2016). However, this theoretical framework inadequately explains the early initiation, self-perpetuating nature, and irreversibility characteristic of the degenerative process (López-Otín et al., 2023). Although accumulating evidence suggests profound biological associations between IDD and organismal aging, the mechanisms underlying the “programmatic” decline of cellular function with advancing age in intervertebral discs—tissues characterized by low cellular turnover—remain enigmatic (Horvath and Raj, 2018).

Recent advances in aging biology and epigenetics have provided novel insights into this fundamental question. Accumulating evidence indicates that cellular function extends beyond the classical “Hayflick limit” of replicative senescence, instead being governed by an intrinsic “epigenetic clock” that precisely modulates cellular states (Zhang et al., 2023). This clock orchestrates gene expression patterns through heritable chemical modifications, including DNA methylation and histone modifications, thereby maintaining cellular “youthfulness” and normal physiological function (Aunan et al., 2017). Accordingly, we propose a central hypothesis: IDD fundamentally represents an accelerated dysregulation of the epigenetic clock within local intervertebral disc tissues (Coppé et al., 2010). Such dysregulation actively suppresses critical programs governing cellular homeostasis and matrix synthesis, while simultaneously initiating a destructive microenvironment characterized by the senescence-associated secretory phenotype (SASP) (Gradisnik et al., 2025), ultimately culminating in structural disintegration and the emergence of clinical symptoms.

A critical epistemological question must be acknowledged: are the observed epigenetic changes in IDD drivers or passengers? We propose a “vicious cycle” model wherein initial mechanical/oxidative stress triggers early epigenetic silencing (e.g., SOX9 promoter hypermethylation) that initiates senescence, while SASP factors further propagate epigenetic dysregulation in neighboring cells (Li et al., 2025). Resolving causality requires longitudinal *in vivo* tracking of epigenetic marks coupled with CRISPR-dCas9-based locus-specific editing.

Based on this paradigm shift, this review aims to synthesize recent advances in the field and construct a coherent conceptual framework spanning from molecular mechanisms to therapeutic strategies. We first distinguish the heterogeneity of senescent cell subsets in IDD and resolve the core controversies in the field, then systematically analyze the synergistic regulatory network of epigenetics in disc senescence, and finally propose a staged synergistic treatment strategy based on the current research bottlenecks. Through this systematic discussion, we seek to elucidate a fundamental conceptual evolution: the therapeutic objective in IDD is transitioning from passive retardation of structural degradation toward active reprogramming of cellular fate and reversal of biological age, thereby establishing a theoretical foundation for functional intervertebral disc regeneration.

## 2 Cellular senescence in IDD: complex phenotypes and a permissive microenvironment

IDD has long been conceptualized as a passive “wear and tear” process. However, emerging evidence has fundamentally revised this view, revealing IDD as a progressive pathological condition actively driven by cellular biological events, with cellular senescence serving as a central pathogenic driver (Silwal et al., 2023). Rather than undergoing apoptosis, senescent disc cells persist in a dysfunctional “zombie-like” state—they not only lose their capacity for proliferation and synthesis of healthy extracellular matrix (ECM), but also actively perturb the surrounding microenvironment through secretion of deleterious factors. This process establishes a self-sustaining and progressively expanding vicious cycle that orchestrates the initiation and progression of IDD (Jeon et al., 2017) (Table 1).

**TABLE 1:** Cellular and microenvironmental senescence in IDD: Key mediators and therapeutic implications.

Anatomical compartment	Primary senescence inducers	Core pathway(s)	Key pathological consequences	Potential intervention(s)	References
Nucleus Pulposus (NP)	Cyclic mechanical tension; High-magnitude compression; High glucose; AGE accumulation	p53-p21-Rb; p38-MAPK; Piezo1	Proteoglycan loss; NP dehydration; Impaired cell viability	N-cadherin overexpression; Autophagy activation; Metformin	Rider et al. (2018), Vadalà et al. (2019), Shi et al. (2022), Feng C. et al. (2018), Tsujimoto et al. (2022), Shao et al. (2021), Zhang X. et al. (2024), Niu et al. (2018), Li F. et al. (2024)
Annulus Fibrosus (AF)	High mechanical stretch; Aberrant mechanical stress	RhoA/ROCK; p38-MAPK; Oxidative stress	Collagen disruption; Loss of structural integrity; Spinal instability	RhoA/ROCK pathway inhibitors; Antioxidants; Senolytics	Feng et al. (2017), Li S. et al. (2024), Ning et al. (2021), Meng Z. et al. (2025)
Cartilage Endplate (CEP)	Inflammation; Nutrient deprivation	IL-1β/TNF-α signaling; Metabolic dysregulation	Calcification; Impaired nutrient transport to NP	Anti-inflammatory therapy; Permeability modulation	Silwal et al. (2023), Vadalà et al. (2019), Yang et al. (2024)
Disc Microenvironment (global)	Oxidative stress; Immune infiltration; Metabolic disorders (e.g., diabetes)	ROS-NF-κB; MAPK/JNK axes	Chronic inflammation; Redox imbalance; Accelerated overall degeneration	Glutathione; Immunomodulation; Glycemic control	Silwal et al. (2023), Feng et al. (2017), Li L. et al. (2024), Li S. et al. (2024), Li F. et al. (2024), Li et al. (2025), Ren et al. (2025)

### 2.1 Heterogeneity of senescent cells: beyond nucleus pulposus cells

Conventional perspectives have often attributed intervertebral disc aging primarily to nucleus pulposus (NP) cells. However, studies with single-cell resolution have revealed marked spatiotemporal heterogeneity and functional diversity among senescent cells in IDD (Rohanifar et al., 2022). This heterogeneity manifests across two principal dimensions.

First, cells from distinct anatomical regions exhibit differential susceptibility to senescence and divergent response patterns. NP, annulus fibrosus (AF), and cartilage endplate (CEP) cells—owing to their distinct microenvironments and functional demands—may enter senescence through different molecular pathways (e.g., p53-p21CIP1, p16INK4a-Rb, NF-κB) and display unique SASP profiles (Feng C. et al., 2018; Pattappa et al., 2012). Studies have demonstrated that AF cells are particularly susceptible to aberrant mechanical stress, with excessive loading directly activating the p38 MAPK-p16 pathway to induce stress-induced premature senescence (Yang et al., 2025). In contrast, senescence of CEP cells closely correlates with nutrient channel obstruction and cumulative oxidative stress, manifesting as prominent mitochondrial dysfunction (Zhang S.‐P. et al., 2024). Recent single-cell transcriptomic atlases have begun to quantitatively refine the regional distinctions among intervertebral disc compartments. Gan et al. mapped the transcriptomic landscape of 108,108 IVD cells from young and adult healthy discs across NP, AF, and CEP, classifying chondrocyte subclusters based on their regulatory, homeostatic, and effector functions in extracellular matrix homeostasis (Gan et al., 2021). Wang et al. subsequently profiled NP and AF cells together with immunocytes across different stages of degeneration (Pfirrmann grades I–V), identifying six NP subclusters and seven AF subclusters with distinct functional characteristics and distribution patterns during disease progression (Wang J. et al., 2021). At the progenitor level, a PROCR^+^ resident progenitor population was identified in healthy NP with enriched colony-forming unit-fibroblast activity and trilineage differentiation capacity, while MCAM^+^ progenitors were identified in AF and CD24^+^/MKI67^+^ progenitors in NP (Gan et al., 2021; Wang J. et al., 2021). Furthermore, Swahn et al. performed scRNA-seq on surgically separated AF (19,978 and 26,983 cells) and NP (20,884 and 24,489 cells) from healthy and diseased human IVDs, revealing both shared and compartment-specific degenerative processes—including depletion of immature fibroblast progenitors and stem cells in both tissues, expansion of fibrotic populations in degenerated NP, and a novel disease-associated subset in degenerated AF expressing pathogenic genes (Swahn et al., 2024). These spatially resolved single-cell datasets collectively establish that NP, AF, and CEP cells exhibit fundamentally distinct transcriptomic programs in both health and degeneration, providing a quantitative framework for developing compartment-targeted therapeutic strategies.

**FIGURE 1:**
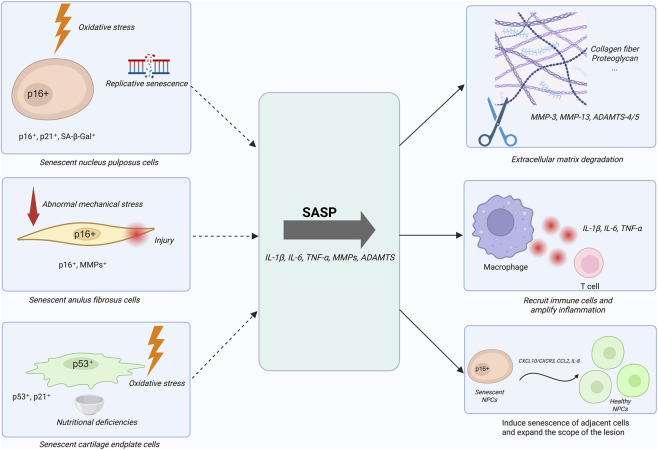
Heterogeneity of cellular senescence in intervertebral disc degeneration and its pathological contributions. The intervertebral disc comprises three anatomically and functionally distinct compartments—nucleus pulposus (NP), annulus fibrosus (AF), and cartilage endplate (CEP)—each exhibiting differential susceptibility to senescence induction and distinct senescence-associated secretory phenotype (SASP) profiles. NP cells predominantly senesce via p53-p21 pathway activation and secrete matrix-degrading enzymes (ADAMTS4/5, MMP13), contributing to proteoglycan loss and dehydration. AF cells activate p38 MAPK-p16^INK4a^ signaling under mechanical stress and produce chemokine-dominant SASP (CCL2, CXCL1), promoting structural disorganization. CEP senescent cells exhibit mitochondrial dysfunction and secrete angiogenic/osteogenic factors (VEGFA, BMP2), leading to calcification and impaired nutrient diffusion. A pathogenic fibrotic NP subpopulation (Fibro-NPC) identified by single-cell sequencing functions as a terminal disease-driving subtype. These compartment-specific senescence programs collectively establish a self-amplifying degenerative ecosystem (Created by the Biorender).

Second, even within the same NP tissue, functionally distinct senescent cell subpopulations coexist. For instance, a fibrotic NP cell subset (Fibro-NPC) identified in 2023 through single-cell sequencing is considered a terminally differentiated pathogenic subtype (Tu et al., 2021). These cells highly express SFRP4, which not only accelerates senescence progression in both themselves and adjacent cells but also directly drives fibrotic changes, vascular invasion, and inflammatory infiltration within the NP. Thus, Fibro-NPCs have emerged as a critical link connecting cellular senescence with structural tissue deterioration (Shao et al., 2016). This finding suggests that targeted intervention against specific pathogenic senescent subpopulations may offer greater clinical value than non-discriminatory “anti-aging” strategies (Figure 1).

### 2.2 Senescence-associated secretory phenotype: the engine of a permissive microenvironment

The SASP constitutes the principal instrument through which senescent cells influence their surrounding environment, comprising a heterogeneous cocktail of pro-inflammatory cytokines, matrix-degrading enzymes, growth factors, and other bioactive molecules (Kumari and Jat, 2021). In the context of IDD, the SASP functions not as innocuous background noise but rather as an active destructive engine that drives degenerative progression, with its pathogenic effects manifesting across three interrelated levels.

#### 2.2.1 Direct extracellular matrix degradation

SASP components include significantly upregulated matrix metalloproteinases (e.g., MMP-3, MMP-13) and aggrecanases (e.g., ADAMTS-4/5) (Horváth et al., 2023). These enzymes function as “molecular scissors,” efficiently degrading type II collagen and aggrecan—the core constituents responsible for maintaining disc hydration and elasticity—thereby directly compromising intervertebral disc structure and function (Ashruf and Ansari, 2022). It has been shown that conditioned medium from senescent NP cells contains elevated MMP-13 activity, which is capable of degrading a major fraction of aggrecan over a short time course (Tilotta et al., 2023; Li Z. et al., 2023).

#### 2.2.2 Immune cell recruitment and chronic inflammation induction

SASP factors, including potent pro-inflammatory cytokines such as interleukin-1β (IL-1β), IL-6, and tumor necrosis factor-α (TNF-α), establish a persistent chronic inflammatory microenvironment (Tylutka et al., 2024). This not only exacerbates pain perception but also activates pro-inflammatory signaling pathways, including NF-κB, in additional cells, thereby establishing a self-amplifying positive feedback loop of inflammation (Chung et al., 2018). For instance, IL-1β has been shown to inhibit chaperone-mediated autophagy (CMA), consequently driving more cells toward senescence (Silwal et al., 2023). Clinical investigations have revealed that IL-6 levels in degenerated disc tissue correlate positively with patient pain scores (Johnson et al., 2015).

#### 2.2.3 Induction of “paracrine senescence”

This represents the most critical pathogenic feature of the SASP. SASP factors can diffuse to and act upon neighboring, previously healthy disc cells, inducing senescence in these cells through paracrine signaling (Acosta et al., 2013). This process resembles “transmission,” leading to exponential expansion of the senescent cell population within the tissue and rapidly depleting its regenerative capacity—thus explaining the progressive and irreversible nature of IDD (Stojanović et al., 2025). Mechanistic studies have identified the CXCL10/CXCR3 axis as a key signaling pathway mediating this paracrine senescence effect: CXCL10 secreted by senescent cells activates the p38 MAPK pathway in adjacent cells, upregulating p16 expression and inducing senescence (Morein et al., 2020). Additionally, chemokines including CCL2 and IL-8 have been implicated in this process (Cao et al., 2025).

### 2.3 Interplay between microenvironmental stress and senescence: a vicious cycle

The uniquely harsh microenvironment of the intervertebral disc—characterized by hypoxia, nutrient deprivation, low pH, and elevated osmotic pressure—serves both as an instigator of senescence and as a condition exacerbated by senescent cells, thereby establishing a tightly coupled bidirectional vicious cycle (Hechavarria and Richard, 2022).

#### 2.3.1 Stress-induced senescence

Aberrant mechanical stress, such as excessive compression, activates Piezo1 ion channels, triggering reactive oxygen species bursts and DNA damage, which in turn activate the p38 MAPK-p53-p21 pathway and culminate in stress-induced premature senescence (SIPS) (Silwal et al., 2023; Guo et al., 2023; Liu et al., 2022). Recent investigations have identified the ROCK-ERK1/2 signaling cascade as a critical downstream pathway mediating mechanical stress-induced senescence in NP cells (Ning et al., 2021). Oxidative stress and genotoxic insults directly damage DNA, activating the canonical p53-p21 pathway through ATM/ATR kinases (Resseguie et al., 2015). Sustained reactive oxygen species leakage resulting from mitochondrial dysfunction is considered a core mechanism driving the transition from replicative to stress-induced senescence (Ye et al., 2020). Acidic conditions, such as pH 6.5, have been shown to induce endoplasmic reticulum stress and promote cell cycle arrest along with senescence marker expression in NP cells (Luo et al., 2023). Nutrient deprivation, particularly glucose and glutamine deficiency, induces cellular senescence through dysregulation of the AMPK-mTOR signaling axis (López et al., 2019).

#### 2.3.2 Senescence-mediated microenvironmental deterioration

Senescent cells, through the abundant inflammatory factors and proteases released via the SASP, further compromise ECM integrity, alter tissue biomechanical properties, and potentially impair already tenuous nutrient diffusion pathways (Vo et al., 2016; Rider et al., 2018). Concurrently, senescence-associated metabolic reprogramming, including enhanced glutamine metabolism, may alter local metabolite concentrations, exacerbating microenvironmental acidification or nutrient competition (Zhang F. et al., 2024). This positive feedback mechanism renders the degenerative process refractory to reversal once initiated.

Recent investigations have identified key regulators of this vicious cycle. For instance, chaperone-mediated autophagy (CMA) deficiency has been established as a central link connecting inflammatory stress with cellular senescence (Silwal et al., 2023). Impaired CMA function leads to intracellular accumulation of DYRK1A protein, which subsequently drives cell cycle arrest and senescence (Cheng Z. et al., 2025). Notably, CMA deficiency concurrently enhances glutamine metabolism in senescent cells, conferring enhanced survival advantages through anti-apoptotic mechanisms and rendering these cells more resistant to clearance (Cheng Z. et al., 2025). These findings provide novel insights into the mechanisms by which senescent cells persist within and exert deleterious effects upon the harsh disc microenvironment.

Furthermore, aberrant expression of epigenetic regulators such as BRD4 has been implicated in promoting both NPC senescence and ECM degradation through upregulation of the MAP2K7/PGF signaling axis, establishing another signaling hub that facilitates IDD progression (Zhang G. et al., 2025). BRD4 inhibitors, including JQ1, have demonstrated efficacy in attenuating the senescent phenotype of NP cells and restoring matrix synthesis capacity *in vitro* (Hong et al., 2020).

**FIGURE 2:**
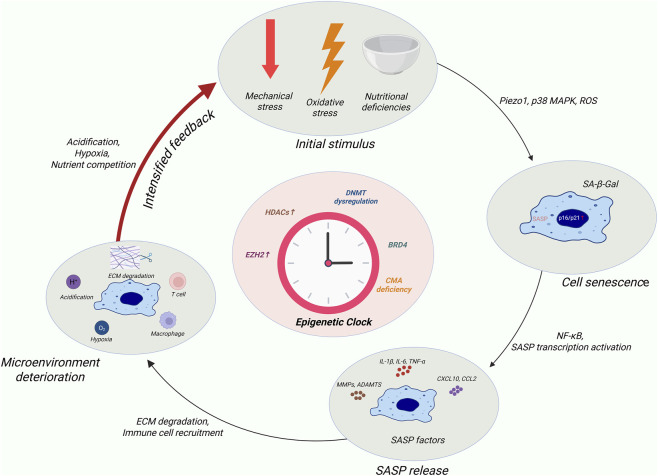
The vicious cycle of senescence, epigenetic dysregulation, and microenvironmental deterioration in IDD. Mechanistic and metabolic stressors—including mechanical overload, oxidative stress, nutrient deprivation, and acidic pH—initiate cellular senescence through Piezo1 channel activation, ROS bursts, and DNA damage. Senescent cells, via their SASP, directly degrade the extracellular matrix, recruit immune cells, and induce paracrine senescence in neighboring healthy cells, thereby expanding the senescent population. Key amplifiers of this cycle include chaperone-mediated autophagy (CMA) deficiency, which stabilizes DYRK1A and enhances glutamine-dependent senescent cell survival, and epigenetic reader BRD4, which sustains SASP gene expression. This self-perpetuating loop renders the degenerative process progressively refractory to homeostatic correction (Created by the Biorender).

In summary, cellular senescence in IDD represents a highly heterogeneous and actively aggressive process. Through the pleiotropic “arsenal” of the SASP, local stress signals are amplified into sustained tissue-wide cascades of destruction, establishing a self-perpetuating and refractory microenvironment. Targeting critical nodal points within this complex network—including specific SASP components, CMA, and BRD4—has emerged as a Frontier strategy for disrupting this vicious cycle and achieving disease-modifying therapy (Silwal et al., 2023; Sun J. et al., 2023; Nadeem et al., 2025) (Figure 2).

## 3 Epigenetic regulatory networks: the molecular gears driving the aging clock

If cellular senescence represents the driver of the IDD “vehicle,” then epigenetic regulatory networks constitute the intricate molecular machinery that propels it. Epigenetics encompasses mechanisms that regulate gene expression through chemical modifications and chromatin remodeling without altering the underlying DNA sequence. Within the intervertebral disc, dysregulation and mistiming of these “molecular gears” directly precipitate the loss of NP cell identity and the initiation of senescent programs. Three principal regulatory modules, including DNA methylation, histone modifications, and non-coding RNAs, synergistically or antagonistically interact to accelerate the epigenetic clock of the intervertebral disc.

### 3.1 DNA methylation: global orchestrator of gene silencing

DNA methylation represents the earliest identified and most extensively characterized epigenetic modification. This modification involves the addition of methyl groups to cytosine residues within CpG dinucleotides, typically resulting in transcriptional gene silencing (Nan et al., 1998).

#### 3.1.1 Coexistence of global hypomethylation and locus-specific hypermethylation

During IDD progression, DNA methylation patterns exhibit complex bidirectional alterations. On one hand, the genome displays global hypomethylation. This widespread loss of methylation may induce genomic instability and activate retrotransposons, thereby triggering cellular stress and senescence (Soto-Palm et al., 2022; Zhu et al., 2021). On the other hand, specific gene promoter regions manifest aberrant hypermethylation. Studies have confirmed that promoter regions of genes essential for NP cell function—including ACAN, which encodes the major matrix protein aggrecan, and SOX9, a master transcription factor maintaining cell phenotype—exhibit significantly elevated methylation levels in degenerated tissues. This hypermethylation impedes transcription of these genes, consequently diminishing extracellular matrix synthesis capacity (Hu X. et al., 2021; Milheiro et al., 2025).

#### 3.1.2 Direct evidence for “epigenetic age acceleration”

Epigenetic clocks, such as the Horvath clock, have been developed based on DNA methylation patterns to accurately predict the biological age of tissues. A pioneering study examining human intervertebral disc samples revealed that severely degenerated discs exhibited a significantly higher epigenetic age relative to the chronological age of the donors, demonstrating an “age acceleration” phenomenon. Moreover, the extent of this acceleration correlated positively with the grade of degeneration (Bao et al., 2025; Saul and Kosinsky, 2021; Lee et al., 2024). These findings provide compelling molecular evidence supporting the conceptualization of IDD as a localized premature aging process. The key enzymes driving this process include DNA methyltransferases and TET demethylases. Mechanistic investigations have revealed that degenerated NP cells display downregulation of DNMT1, which maintains methylation patterns, alongside upregulation of TET1, which mediates active demethylation. This dysregulation may represent a critical mechanism underlying the aberrant methylation patterns observed in IDD (Liu R. et al., 2023; Kong et al., 2025; Hou et al., 2026).

The question of whether epigenetic clock dysregulation is causal in IDD remains largely unresolved, as direct evidence from longitudinal and interventional studies is exceedingly sparse. Circumstantial support derives from three directions. First, cross-sectional human studies have established that promoter hypermethylation of SOX9 and ACAN correlates with degeneration grade and reduced matrix gene expression, though temporal precedence has not been demonstrated. Second, the senolytic combination dasatinib plus quercetin (D+Q) reduces disc senescent cell burden and ameliorates age-dependent structural degeneration in mice, but whether this is accompanied by reversal of epigenetic clock CpG methylation in disc tissues remains unexamined; a separate longitudinal study of D+Q in non-disc contexts found limited effects on DNA methylation clocks (Lee et al., 2024; Novais et al., 2021). Third, the DNMT inhibitor 5-azacytidine protects NP cells from IL-1β-induced apoptosis and endoplasmic reticulum stress by preserving PPARγ via promoter demethylation, providing proof-of-concept that pharmacological DNA demethylation can confer functional benefits—though restoration of SOX9 or other master NPC identity genes has not been directly shown (Cheng P. et al., 2025). Resolving causality will require dedicated longitudinal multi-omics studies that track epigenetic marks at serial timepoints in animal models, coupled with interventional epigenome editing.

Furthermore, existing epigenetic clocks have limited generalizability to IDD. Most widely used clocks (including the Horvath clock) were trained primarily on peripheral blood and mitotically active tissues, with minimal representation of avascular, low-turnover intervertebral disc samples. Bulk tissue methylation analysis also averages signals across heterogeneous cell populations, obscuring the pronounced epigenetic age acceleration specific to senescent cells. These methodological limitations complicate cross-sectional study interpretation and hinder biomarker development. A disc-specific epigenetic clock trained on single-cell resolution data is therefore a prerequisite for validating the epigenetic clock hypothesis in IDD.

### 3.2 Histone modifications: dynamic switches governing chromatin state

Histones function as spools around which DNA is wrapped, with their N-terminal tails undergoing diverse chemical modifications—including methylation, acetylation, and phosphorylation. These modifications collectively constitute the “histone code,” precisely orchestrating the open or closed configuration of chromatin (Tessarz and Kouzarides, 2014).

#### 3.2.1 The disrupted balance between “writing” and “erasing”

In IDD, a profound imbalance emerges between histone modifications that repress gene transcription and those that promote transcriptional activation (Liu R. et al., 2023).

##### 3.2.1.1 Accumulation of repressive modifications

EZH2, a core component of the Polycomb Repressive Complex 2, catalyzes the trimethylation of histone H3 at lysine 27 (H3K27me3)—a potent transcriptional repression mark. Multiple studies have demonstrated that EZH2 expression is significantly upregulated in degenerated NP tissues, leading to its enrichment at promoter regions of genes critical for NP cell identity, including FOXF1 and KRT19. This enrichment effectively “locks” these genes in a repressed state, thereby promoting cellular dedifferentiation (Deng et al., 2020; Zhu et al., 2025; Zhu et al., 2024; Yao et al., 2024). Treatment of degenerated cells with EZH2 inhibitors effectively reduces H3K27me3 levels (Egan et al., 2016).

##### 3.2.1.2 Loss of activating marks

Histone acetylation, typically associated with open chromatin architecture and active transcription, is catalyzed by histone acetyltransferases, whereas histone deacetylases (HDACs) remove acetyl groups and suppress transcription. Investigations have revealed that HDAC family members exhibit aberrantly elevated activity in IDD, resulting in excessive deacetylation and consequent silencing of chromatin regions harboring key repair genes (Milazzo et al., 2020; Shukla and Tekwani, 2020). Furthermore, BET family proteins, which function as “readers” of acetylation signals, play essential roles in mediating transcriptional elongation of inflammatory genes. Inhibitors targeting BET proteins have demonstrated anti-inflammatory and cartilage degeneration-slowing effects in osteoarthritis models, and their potential roles in IDD are emerging as active research frontiers (Wijnen, 2019; Wang N. et al., 2021; Sun M. et al., 2023).

#### 3.2.2 Crosstalk between histone modifications and DNA methylation

These two epigenetic mechanisms do not function in isolation but rather engage in extensive crosstalk. For instance, EZH2-mediated H3K27me3 can recruit DNA methyltransferases, thereby converting repressive histone marks into more stable DNA methylation. Conversely, DNA methylation within specific genomic regions can influence the modification status of adjacent histones. This synergistic interplay establishes an “epigenetic silencing complex,” rendering degeneration-associated gene expression programs exceptionally stable and refractory to reversal once established (Zhang et al., 2015; Voigt et al., 2013).

### 3.3 Non-coding RNAs: network hubs of fine-tuned regulation

Long non-coding RNAs and microRNAs constitute a third dimension of epigenetic regulation. These molecules function both as upstream signals that modulate epigenetic modifying enzymes and as downstream effectors that transduce regulatory signals (Wang M. et al., 2018; Wang et al., 2019).

#### 3.3.1 MiRNAs: fine-tuners of degenerative progression

Numerous miRNAs exhibit dysregulated expression in intervertebral disc degeneration (IDD), actively participating in key pathological processes including NP cell apoptosis, inflammation, extracellular matrix degradation, and AF degeneration. For instance, miR-138-5p promotes TNF-α-induced apoptosis via the PTEN/PI3K/Akt pathway, while miR-27a enhances inflammatory cytokine release through p38/MAPK signaling. Additionally, miR-27b accelerates matrix degradation by targeting MMP13, and miR-221 loss contributes to AF osteogenic differentiation. Notably, certain miRNAs (e.g., miR-30d, miR-665) concurrently regulate multiple degenerative pathways, highlighting their potential as therapeutic targets for IDD (Wang C. et al., 2022).

#### 3.3.2 lncRNAs and circRNAs: molecular scaffolds for epigenetic modification

Long non-coding RNAs and circular RNAs frequently function as “molecular sponges” that adsorb miRNAs, thereby relieving the repression of target genes and exerting regulatory effects. More importantly, certain lncRNAs can directly serve as “scaffolds” that recruit chromatin-modifying complexes to specific genomic loci. For example, lncRNA HOTAIR simultaneously binds both the PRC2 complex and the LSD1 complex, enabling dual epigenetic repression at individual gene loci (Yang et al., 2016; Zhang et al., 2019). Studies have demonstrated that HOTAIR is highly expressed in IDD tissues, potentially contributing to the epigenetic silencing of matrix synthesis genes (Hou et al., 2026; Guo et al., 2020). Additionally, study demonstrates that exosomal circRNA_0000253 promotes intervertebral disc degeneration by sponging miR-141-5p and downregulating SIRT1, leading to increased NP cell apoptosis and extracellular matrix degradation. Silencing circRNA_0000253 or restoring miR-141-5p effectively alleviates IDD progression (Song et al., 2020).

#### 3.3.3 An integrated ncRNA-epigenetic regulatory network in IDD

These diverse ncRNAs collectively form a regulatory network operating through two principal modes: chromatin-level scaffolding and post-transcriptional ceRNA crosstalk. At the chromatin level, HOTAIR serves as a modular scaffold that simultaneously binds the PRC2 and LSD1/CoREST/REST complexes, thereby coordinating H3K27 methylation and H3K4 demethylation at target gene loci (Tsai et al., 2010). In IDD, HOTAIR is upregulated in degenerative NP tissues, where it promotes senescence, apoptosis, and ECM degradation via the Wnt/β-catenin pathway (Zhan et al., 2019), and independently functions as a ceRNA to sponge miR-148a, upregulating PTEN and further driving autophagy and apoptosis (Zhang S. et al., 2022). At the competitive RNA level, exosomal circRNA_0000253, the most highly upregulated circRNA in degenerative NP-derived exosomes, adsorbs miR-141-5p to downregulate SIRT1 (Song et al., 2020), while circ-TIMP2 sponges miR-185-5p to upregulate MMP2, thereby promoting ECM catabolism (Guo et al., 2020). This interconnected ncRNA network converges on the silencing of NPC identity genes (e.g., SOX9, ACAN, KRT19) (Lv et al., 2016) and the concurrent activation of matrix-degrading enzymes and inflammatory mediators (Guo et al., 2020), representing a multi-node intervention opportunity.

### 3.4 mTOR signaling: a metabolic-epigenetic hub linking nutrient status, autophagy, and senescence

The mechanistic target of rapamycin (mTOR), a serine/threonine kinase that integrates nutrient and growth factor signals, functions as a central regulator of cell growth, autophagy, and senescence (Ito et al., 2017; Yurube et al., 2024). mTOR operates via two distinct complexes: mTORC1, which promotes anabolic processes and suppresses autophagy, and mTORC2, which regulates cell survival and cytoskeletal organization (Laplante et al., 2012). In the context of IDD, accumulating evidence positions mTORC1 as a key pathogenic driver (Ito et al., 2017; Yurube et al., 2024).

mTORC1 links autophagy suppression to senescence. mTORC1 directly phosphorylates and inactivates ULK1 (at Ser757 and Ser777) and ATG13, core components of the autophagy initiation complex, thereby suppressing autophagic flux (Kim et al., 2011; Hosokawa et al., 2009). Under nutrient-deprived conditions, this mTORC1-dependent phosphorylation is relieved, permitting autophagy initiation. In the intervertebral disc—an avascular, nutrient-poor organ—this regulatory axis is particularly relevant. Impaired autophagy leads to the accumulation of damaged mitochondria, ROS, and misfolded proteins, which collectively accelerate cellular senescence. Autophagy restoration through mTORC1 inhibition has been shown to rescue oxidative damage and protect against disc degeneration (Ito et al., 2017; Yurube et al., 2024).

mTOR selectively promotes the SASP at the translational level. A seminal study by Laberge et al. demonstrated that mTORC1 inhibition by rapamycin suppresses the SASP through a specific translational mechanism: rapamycin reduces the translation of membrane-bound IL-1α, which in turn diminishes NF-κB transcriptional activity—the master regulator of numerous SASP factors (Laberge et al., 2015). Notably, rapamycin reduced IL-6 and other cytokine mRNA levels, but its primary effect was at the translational level of IL-1α; exogenous IL-1α restored IL-6 secretion in rapamycin-treated senescent cells (Laberge et al., 2015). These findings establish a direct mechanistic link between metabolic sensing by mTOR and the inflammatory microenvironment characteristic of cellular senescence.

mTOR-engaged crosstalk with the epigenetic machinery. mTOR signaling exhibits extensive crosstalk with the epigenetic machinery, though the directionality is often bidirectional. mTORC1 has been shown to directly phosphorylate and activate the histone acetyltransferase p300, thereby regulating autophagy and lipogenesis (Wan et al., 2017). Conversely, p300 can acetylate raptor, a core component of mTORC1, modulating mTORC1 activity (Son et al., 2024). Furthermore, mTOR modulation induces selective perturbations in histone methylation—notably H3K27me3—through distinct mechanisms depending on whether mTOR is activated or inhibited (Kim et al., 2024). These findings collectively indicate substantial interplay between mTOR signaling and the epigenetic landscape, though the precise consequences for SASP gene expression in the context of IDD remain to be fully elucidated.

### 3.5 Remodeling of three-dimensional chromatin architecture

Ultimately, all the aforementioned epigenetic marks influence the three-dimensional spatial conformation of chromatin within the nucleus. Topologically associating domains (TADs) serve as fundamental structural units of the genome, within which fine-tuned gene regulation is achieved through enhancer-promoter looping. Emerging evidence suggests that during degenerative progression, alterations in matrix stiffness and abnormalities in nuclear scaffold proteins may lead to loosening of TAD structures associated with cell identity maintenance, accompanied by disruption of critical enhancer-promoter loops. Such architectural disorganization could precipitate global misexpression of cell identity genes (Dekker and Mirny, 2024; Mohanta et al., 2021; Maurer et al., 2019). Although investigations into this dimension of epigenetic dysregulation in IDD remain in their infancy, this area undoubtedly represents a critical Frontier for future understanding of epigenetic reprogramming mechanisms.

**FIGURE 3:**
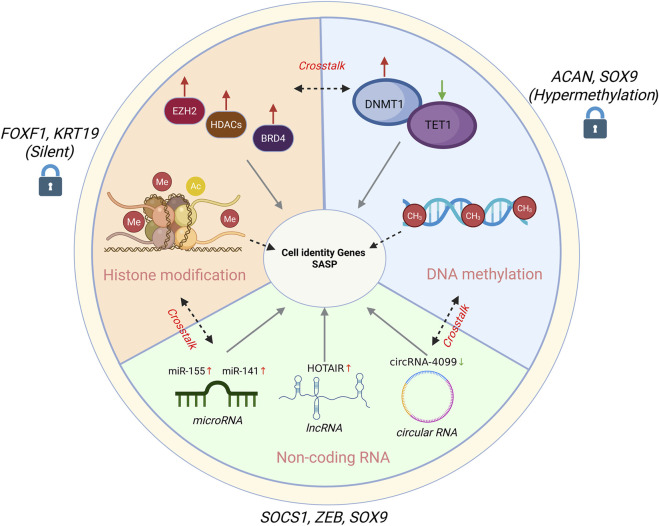
Epigenetic regulatory networks driving intervertebral disc degeneration. Three interconnected layers of epigenetic control converge to drive IDD: (1) DNA methylation—global hypomethylation induces genomic instability while locus-specific hypermethylation at SOX9, ACAN, and FOXF1 promoters silences NPC identity genes; DNMT1 downregulation and TET1 upregulation underlie these bidirectional changes; (2) Histone modifications—EZH2-mediated H3K27me3 accumulation represses key NPC genes, while aberrant HDAC activity deacetylates chromatin at repair loci; BET proteins read acetylation marks to drive inflammatory transcription; extensive crosstalk between H3K27me3 and DNA methylation establishes a stable epigenetic silencing complex (3) Non-coding RNAs—lncRNAs (e.g., HOTAIR); serve as molecular scaffolds recruiting PRC2 and LSD1 to target loci, while exosomal circRNAs (e.g., circRNA_0000253) sponge protective miRNAs to promote apoptosis and matrix degradation. These layers collaboratively remodel the three-dimensional chromatin architecture, disrupting topologically associating domains (TADs) and enhancer-promoter loops essential for NPC identity (Created by the Biorender).

In summary, DNA methylation, histone modifications, and non-coding RNAs constitute three interconnected regulatory layers that culminate in a highly intricate network. In IDD, this network undergoes systemic dysregulation: not only are genes essential for maintaining youthful cellular function silenced, but destructive inflammatory and senescent programs are simultaneously activated, collectively precipitating the inexorable progression of disc cells toward degenerative fate (Kang et al., 2023; Li G. et al., 2022) (Figure 3).

## 4 Therapeutic strategies: from senescent cell clearance to cellular fate reprogramming

The reconceptualization of IDD as a cellular senescent program driven by epigenetic dysregulation has precipitated a profound paradigm shift in therapeutic approaches. Traditional anti-inflammatory or growth factor supplementation strategies resemble attempts to repair a vehicle whose engine has already ignited, whereas emerging interventions directly target the pathological nidus: either eliminating the already “combusting” senescent cells, reactivating cellular vitality through modulation of epigenetic “master switches,” or completely “replacing the engine” via reprogramming technologies to restore youthful cellular identity (Zhang et al., 2020; Liu Yang et al., 2024). This section systematically examines the research progress and translational prospects of these Frontier strategies, organized according to their depth of intervention.

It must be emphasized that essentially all of these strategies remain in preclinical development; no epigenetic or senolytic therapy has entered clinical trials for IDD. The following sections therefore critically balance therapeutic promise against substantial translational hurdles.

### 4.1 Senolytics: precision clearance of senescent cells

Senolytics constitute a class of pharmacological agents that selectively induce apoptosis in senescent cells. The theoretical foundation underlying this approach rests on the observation that senescent cells have evolved anti-apoptotic pathways to resist cell death within harsh microenvironments—a dependency that creates an exploitable vulnerability (Kirkland and Tchkonia, 2020; Zhu et al., 2015).

#### 4.1.1 Classic combination: dasatinib plus quercetin

Dasatinib, a tyrosine kinase inhibitor, effectively targets senescent fibroblasts and pre-adipocytes, whereas quercetin, a natural flavonoid compound, demonstrates efficacy against senescent human endothelial cells and mouse bone marrow stem cells. The combination of these two agents represents the most extensively studied senolytic regimen (Power et al., 2023). In animal models of IDD, *D+Q* treatment has been demonstrated to effectively clear senescent cells within the NP, reduce SASP factor levels, increase proteoglycan content, and partially restore disc height. Intraperitoneal administration of *D+Q* attenuated tail puncture-induced intervertebral disc degeneration in mice and improved pain-associated behavioral manifestations (Novais et al., 2021).

#### 4.1.2 Next-generation senolytics

Fisetin, another natural flavonoid compound, exhibits even greater senolytic potency than quercetin while maintaining a favorable safety profile. Recent investigations have confirmed that fisetin attenuates the senescent phenotype of human NP cells and delays degenerative progression in rat IDD models (Vedova et al., 2025). Navitoclax, a BH3 mimetic, functions through inhibition of BCL-2 family anti-apoptotic proteins. Although highly potent in senescent cell clearance, its clinical application may be limited by on-target toxicity including thrombocytopenia; thus, local delivery strategies represent a viable direction for IDD applications (Paganella et al., 2025; Lérida Viso, 2023). FOXO4-DRI, a peptide that interferes with FOXO4-p53 interaction, selectively induces apoptosis in senescent cells without affecting proliferating cells. While research on its application in IDD remains in early stages, its mechanistic selectivity has garnered substantial interest (Xiang H. et al., 2025; Rabinovitch, 2023).

Three additional translational challenges remain unaddressed. First, pharmacokinetic mismatch: rodent studies use high-dose systemic administration, but human IDD requires local intradiscal injection. The human NP (1–2 mL volume) severely limits single-injection dose, while high intradiscal pressure results in drug half-lives of only hours. Second, no non-invasive method exists to quantify senescent cell burden, preventing patient stratification and objective treatment monitoring. Third, non-selective clearance may eliminate residual matrix-synthetic function in early degeneration, potentially exacerbating tissue dysfunction in some patients.

### 4.2 Senomorphics: taming rather than eliminating

In contrast to senolytics, senomorphic agents aim to suppress SASP secretion without eliminating the senescent cells themselves. This strategy offers the advantage of potentially preserving certain beneficial functions that senescent cells may retain while blocking their deleterious effects on the microenvironment (Feng et al., 2025).

#### 4.2.1 mTOR inhibitors

Metabolic-epigenetic modulation of senescence. Rapamycin and its analogs (rapalogs) represent the most extensively characterized senomorphic compounds with direct relevance to IDD. Mechanistically, rapamycin inhibits mTORC1, thereby de-repressing ULK1 to restore autophagic flux, and selectively suppresses IL-1α translation via 4E-BP1-dependent translational control to blunt the SASP (Laberge et al., 2015). In IDD-relevant preclinical models, mTORC1 inhibitors—including rapamycin, temsirolimus, and everolimus—have been shown to protect against inflammation-induced apoptosis, senescence, and extracellular matrix catabolism in human NP cells, with protection dependent on Akt and autophagy induction (Ito et al., 2017). Rapamycin also mitigates IL-1β-mediated disc matrix homeostatic imbalance by inhibiting mTORC1 and inducing autophagy through the PI3K/Akt/mTOR pathway (Yurube et al., 2024). Additionally, glutamine metabolism, which feeds mTORC1 activation through anaplerosis, is increasingly recognized as a modulator of cellular senescence in the disc microenvironment; interruption of glutamine-dependent anaplerosis affects mTORC1/2 activity and can induce a cellular senescence-like response (Liao et al., 2019). This metabolic-epigenetic-senescence interface positions mTOR as a particularly attractive therapeutic target in IDD. Encouragingly, mTOR inhibitors benefit from an extensively characterized safety profile derived from decades of clinical use in transplantation medicine; however, their immunosuppressive effects and the challenge of achieving localized delivery to the avascular disc warrant further optimization (Wu et al., 2022; Davan-Wetton et al., 2021).

#### 4.2.2 JAK inhibitors

The JAK/STAT signaling cascade serves as a common downstream pathway for multiple SASP factors. JAK inhibitors have demonstrated therapeutic potential in senescence-associated chronic inflammatory conditions, and their role in IDD is currently under active investigation (Ren et al., 2025; Liu S. et al., 2023; Hu Xiaoyi et al., 2021).

### 4.3 Limitations and implications

The fundamental limitation of senolytics and senomorphics lies in their mechanism of action: while these agents effectively eliminate dysfunctional cells or suppress their deleterious secretory activity, they cannot replenish the extracellular matrix already degraded nor can they “re-educate” dedifferentiated cells toward a healthy NP phenotype. Consequently, monotherapy employing these strategies alone may prove insufficient to achieve genuine tissue regeneration, leaving an unmet need for deeper-level interventions. This recognition has motivated the exploration of complementary approaches aimed at restoring both cellular identity and synthetic capacity. A comparative summary of all therapeutic strategies including their respective advantages, limitations, and current clinical development stages is provided in Table 2.

**TABLE 2:** Translational status and clinical landscape of emerging therapeutic strategies for intervertebral disc degeneration.

Therapeutic strategy	Representative agents/Approaches	Mechanism of action	Key advantages	Key limitations and risks	Preclinical evidence (IDD models)	Clinical status/Ongoing trials	References
Senolytics	Dasatinib + Quercetin (D+Q); Fisetin; Navitoclax	Selective elimination of senescent cells via targeting anti-apoptotic pathways (SCAPs)	Disease-modifying potential; systemic administration available	Off-target clearance of progenitor cells; transient thrombocytopenia (Navitoclax); limited human IDD data	Attenuated disc degeneration and pain in mouse tail puncture models	Phase I/II trials in osteoarthritis and other age-related conditions; no dedicated IDD clinical trials to date	Power et al. (2023), Novais et al. (2021), Vedova et al. (2025), Paganella et al. (2025)
Senomorphics	Rapamycin; Metformin; JAK inhibitors (Ruxolitinib)	SASP suppression without cell elimination; mTOR or JAK/STAT pathway inhibition	Preserves potentially beneficial senescent cell functions; favorable safety profile	May not remove the underlying senescent cell burden; limited tissue specificity	Reduced SASP and matrix degradation in NPC cultures; Metformin shown to delay IDD in diabetic models	Metformin: extensive clinical safety data; Rapamycin: clinical trials in aging; JAK inhibitors: FDA-approved for inflammatory diseases	Wu et al. (2022), Davan-Wetton et al. (2021), Liu S. et al. (2023), Hu et al. (2021b)
Epigenetic Remodeling (HDAC inhibitors)	Trichostatin A (pan-HDACi); HDAC9-selective inhibitors	Reversal of aberrant histone deacetylation; restoration of matrix gene expression	Broad epigenetic reprogramming potential	Concentration-dependent biphasic effects (pro-survival at low doses, pro-apoptotic at high doses); genome-wide off-target effects	Promoted matrix synthesis in degenerated NPCs at low concentrations; induced apoptosis at high concentrations	Vorinostat, Romidepsin: FDA-approved for hematological malignancies; no IDD trials	Hou et al. (2026), Lei et al. (2023), Ji et al. (2023), Liu et al. (2025)
Epigenetic Remodeling (EZH2 inhibitors)	EPZ-6438 (Tazemetostat); GSK126	Inhibition of H3K27me3 repressive mark deposition; reactivation of silenced NPC identity genes	Specific targeting of a defined epigenetic mark; good target engagement biomarkers (H3K27me3 levels)	Potential impact on immune cell function; long-term effects on genomic stability unknown	Local injection attenuated IDD progression in rat models; reactivated FOXF1 and KRT19 expression	Tazemetostat: FDA-approved for epithelioid sarcoma and follicular lymphoma; no IDD trials	Hou et al. (2026), Yao et al. (2024), Liu et al. (2020), Kordowitzki (2025)
iPSC-Derived Cell Therapy	iPSC-derived notochordal cells; iPSC-derived MSC exosomes	Replacement of lost/dysfunctional NPCs with healthy, youthful cells	Unlimited cell source; potential for autologous transplantation	Teratoma risk from residual undifferentiated cells; phenotypic instability in harsh disc microenvironment; high manufacturing cost	iPSC-derived notochordal cells reduced degeneration in porcine IDD models	Preclinical; no active clinical trials for IDD; multiple iPSC trials for other degenerative conditions	Xiang Q. et al. (2025), Sheyn et al. (2019), Z et al. (2017), Jing et al. (2014)
Direct Lineage Reprogramming	Transcription factor cocktails (e.g., Brachyury, FOXA2); chemical reprogramming	*In situ* conversion of resident fibroblasts or AF cells into NPC-like cells	Bypasses pluripotent intermediate; enables *in situ* regeneration; lower tumorigenicity than iPSCs	Delivery efficiency of reprogramming factors *in vivo*; incomplete reprogramming leading to unstable intermediate states	Conversion of fibroblasts into iNPCs *in vitro*; transplantation slowed degeneration in animal models	Preclinical proof-of-concept; no clinical trials	Wong (2017), Ma et al. (2021), Seki et al. (2022), Tessier and Risbud (2020)
CRISPR-dCas9 Epigenome Editing	dCas9-p300 (activator); dCas9-DNMT3A (silencer)	Locus-specific epigenetic modification without altering DNA sequence	Unprecedented precision; reversible; avoids permanent genetic changes	Delivery efficiency to avascular disc; off-target sgRNA binding; immunogenicity of Cas9 proteins; large cargo size	*In vitro* proof-of-concept in other musculoskeletal tissues; limited IDD-specific studies to date	Preclinical; no clinical trials	Wang et al. (2016), Li T. et al. (2023), Sgro and Blancafort (2020), Jia et al. (2024)

### 4.4 Second front: epigenetic remodeling therapies

If senescent cell clearance represents “housecleaning,” then epigenetic remodeling constitutes “restoring the electrical system of the house.” These strategies aim to correct the epigenetic dysregulation that occurs during IDD progression, reactivating key genes that have been aberrantly silenced and restoring the capacity of cells to synthesize matrix and maintain homeostasis (Stojanović et al., 2025; Alqahtani et al., 2025).

#### 4.4.1 Targeting DNA methylation

##### 4.4.1.1 DNMT inhibitors

5-Azacytidine and decitabine represent classical DNA methyltransferase inhibitors that reverse DNA hypermethylation and reactivate silenced genes. PPARγ methylation mediated by DNA methyltransferase 3a (DNMT3a) promotes intervertebral disc degeneration by activating the NF-κB pathway, leading to increased inflammation and matrix degradation. Conversely, treatment with 5-azacytidine reverses this process by demethylating the PPARγ promoter, thereby preserving PPARγ expression. This preservation inhibits endoplasmic reticulum stress and apoptosis in NP cells, highlighting a potential therapeutic strategy for mitigating disc degeneration through epigenetic regulation (Cheng P. et al., 2025; Cheng et al., 2023). However, DNMT inhibitors lack gene specificity and may induce genome-wide demethylation with potential activation of proto-oncogenes, conferring considerable risk for systemic administration. Therefore, local delivery strategies and low-dose regimens represent critical considerations for clinical translation (Liu R. et al., 2023).

##### 4.4.1.2 Targeting demethylases

TET enzymes mediate active DNA demethylation. Modulating TET enzyme activity, rather than globally inhibiting DNMTs, may represent a more refined approach. However, specific small molecules targeting TET enzymes remain in early developmental stages (Xiao et al., 2020; Wang D. et al., 2022).

Beyond specificity issues, DNA methylation inhibitors face a fundamental biological limitation: their effects are transient and reversible. Pathological methylation patterns will likely re-emerge once treatment is discontinued, necessitating lifelong repeated injections. Global demethylation also carries risks of endogenous retrotransposon activation, genomic instability, and increased cancer risk—a critical concern given the relatively young age of many IDD patients. These challenges confirm that non-specific epigenetic inhibitors cannot serve as standalone therapies and require combination strategies for durable effects.

#### 4.4.2 Targeting histone modifications

##### 4.4.2.1 EZH2 inhibitors

As previously discussed, EZH2 serves as the key enzyme catalyzing the repressive H3K27me3 mark. Multiple studies have confirmed that treatment of degenerated NP cells with EZH2 inhibitors significantly reduces H3K27me3 levels, reactivates NP cell identity genes, and promotes extracellular matrix synthesis (Hou et al., 2026; Yao et al., 2024; Liu et al., 2020; Kordowitzki, 2025). More importantly, local injection of EZH2 inhibitors in animal models has been shown to attenuate intervertebral disc degeneration progression (Yao et al., 2024).

##### 4.4.2.2 HDAC inhibitors

Histone deacetylase inhibitors increase histone acetylation levels and open chromatin architecture. However, their effects in IDD exhibit concentration-dependent duality: at low concentrations, they promote cellular differentiation and matrix synthesis, whereas at high concentrations, they induce apoptosis (Hou et al., 2026; Lei et al., 2023; Ji et al., 2023; Liu et al., 2025). This biphasic effect necessitates precise titration of dosage and administration regimens. Inhibitors selectively targeting specific HDAC subtypes may offer an improved therapeutic window (Zhang et al., 2018).

##### 4.4.2.3 BET inhibitors

BET proteins recognize acetylated histones and facilitate transcriptional elongation, playing particularly critical roles in the expression of pro-inflammatory genes (Wang N. et al., 2021; Wang et al., 2023). BET inhibitors such as JQ1 have demonstrated significant efficacy in suppressing inflammation and attenuating cartilage degeneration in osteoarthritis models (Xu et al., 2024). In NP cells, JQ1 has been confirmed to inhibit IL-1β-induced inflammatory responses and matrix-degrading enzyme expression (Hong et al., 2020).

#### 4.4.3 RNA-based interventions

##### 4.4.3.1 MiRNA mimics/inhibitors

For protective miRNAs that are downregulated during degeneration, supplementation with miRNA mimics may restore their beneficial functions. Conversely, for destructive miRNAs that are upregulated, antagonists such as anti-miRNA oligonucleotides or miRNA sponges can be employed to neutralize their pathological effects (Simonson and Das, 2015). Despite the therapeutic promise of miRNA-based approaches, clinical translation remains hampered by challenges related to delivery efficiency and nucleic acid stability within the target tissue (Wang C. et al., 2022; Holjencin and Jakymiw, 2022).

##### 4.4.3.2 CRISPR-dCas9 epigenetic editing

This technology represents one of the most promising frontiers in epigenetic regulation. By fusing catalytically inactive dCas9 protein with epigenetic modifying enzymes, precise epigenetic modifications at specific genomic loci can be achieved under the guidance of sgRNAs (Wang et al., 2016; Li T. et al., 2023). Theoretically, sgRNAs could be designed to recruit p300 to the SOX9 promoter region, thereby activating its expression through enhanced local acetylation. Alternatively, recruitment of DNMT3A to the MMP13 promoter could silence its expression through targeted methylation (Sgro and Blancafort, 2020). While this technology paints an exciting blueprint for “precision epigenetic therapy” in IDD, current challenges including delivery efficiency and off-target effects must be addressed before clinical translation (Milheiro et al., 2025; Kovalev et al., 2025). A systematic comparison of epigenetic remodeling strategies—including DNMT inhibitors, EZH2 inhibitors, HDAC inhibitors, BET inhibitors, miRNA therapeutics, and CRISPR-dCas9 editing—across key parameters such as target specificity, delivery requirements, and off-target risk profile is presented in Table 2.

### 4.5 Ultimate vision: cellular identity reprogramming

While epigenetic remodeling aims to “repair” existing cells, cellular reprogramming aspires to “create” entirely new, functionally competent youthful cells, which represents the ultimate ambition of regenerative medicine.

#### 4.5.1 Indirect reprogramming: the induced pluripotent stem cell approach

Through reprogramming somatic cells into induced pluripotent stem cells (iPSCs) followed by directed differentiation into NP-like cells, abundant and homogeneous seed cells can be generated for transplantation (Xiang Q. et al., 2025). To date, researchers have successfully directed human iPSCs to differentiate into cells expressing NP markers (Sheyn et al., 2019; Zhu et al., 2017; Jing et al., 2014). However, this approach confronts three major challenges: limited differentiation efficiency, tumorigenic risk associated with residual undifferentiated cells, and the formidable task of maintaining the differentiated phenotype within the harsh *in vivo* microenvironment of the degenerated disc (Xia et al., 2019).

#### 4.5.2 Direct lineage reprogramming: bypassing the stem cell stage

Direct lineage reprogramming refers to the conversion of one terminally differentiated cell type directly into another functional cell type using specific transcription factors or small molecules, without traversing a pluripotent intermediate state (Wong, 2017; Ma et al., 2021). This strategy holds immense potential for *in situ* regeneration within the native tissue environment.

##### 4.5.2.1 Core breakthrough

Through systematic screening, researchers have identified a transcription factor cocktail capable of directly converting mouse fibroblasts into induced NP-like cells (iNPCs) exhibiting characteristic morphology, gene expression profiles, and functional properties of native NP cells (Seki et al., 2022; Tessier and Risbud, 2020). Transplantation of these iPSC-induced notochordal cells into degenerated porcine intervertebral discs significantly slowed the degenerative process (Sheyn et al., 2019).

##### 4.5.2.2 Delivery challenges

The safe and efficient delivery of reprogramming factors to target cells *in vivo* represents the most formidable technical hurdle. Current approaches encompass non-integrating viral vectors, modified mRNA, and chemical reprogramming using small molecule compounds alone (Hamann et al., 2019). Among these strategies, local delivery of reprogramming factor mRNA via injectable hydrogels to achieve *in situ* reprogramming represents a particularly promising direction for future development (Fang et al., 2020). A comparative analysis of indirect (iPSC-based) versus direct reprogramming strategies, including key metrics such as tumorigenic risk, *in vivo* delivery feasibility, and clinical timeline projections, is provided in Table 2.

Direct lineage reprogramming faces three underappreciated translational barriers. First, phenotypic instability: reprogrammed cells often exhibit an immature fetal-like phenotype rather than mature adult nucleus pulposus characteristics, with unproven long-term function in the mechanically active disc microenvironment. Second, aberrant differentiation risk: small variations in reprogramming factor levels can generate fibroblasts or osteoblasts, worsening degeneration or causing pathological calcification. Third, regulatory and efficiency limitations: viral-based *in vivo* reprogramming requires extensive gene therapy safety testing, while non-viral methods currently achieve less conversion efficiency in preclinical models.

### 4.6 A synergistic roadmap

Given the complex, multifactorial nature of IDD, no single intervention is likely to suffice as a definitive therapy. A logical corollary follows: successful future treatments will inevitably comprise multi-target, multi-step combinatorial strategies. Based on this premise, we propose the following “three-step” synergistic treatment roadmap:

Step one: Clearance—elimination of senescent cells. Initial local administration of senolytics would clear the microenvironment of accumulated senescent cells and the deleterious SASP “toxic cloud” they secrete, thereby creating a clean, permissive niche for subsequent therapeutic interventions.
Step two: Priming—epigenetic pre-activation. Subsequent delivery of epigenetic modifiers would globally open chromatin architecture, awakening residual “dormant” NP cells or progenitor populations from epigenetic silencing and rendering them susceptible to reprogramming signals.
Step three: Seeding—reprogramming and regeneration. Finally, introduction of reprogramming factors or functionally competent seed cells into this pre-conditioned microenvironment—already “cleared” and “primed”—would enable authentic *in situ* tissue regeneration.

**FIGURE 4:**
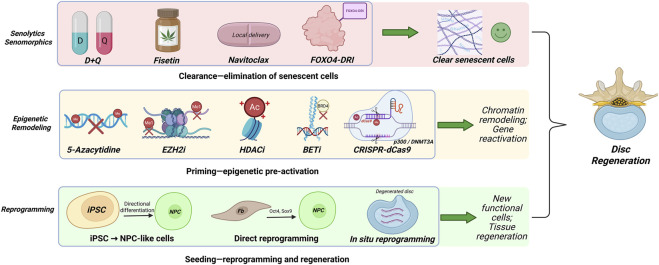
Therapeutic strategies for IDD: from senescent cell clearance to cellular reprogramming. Emerging therapeutic approaches are organized by intervention depth. Senolytics (e.g., Dasatinib + Quercetin, Fisetin) selectively eliminate senescent cells; Senomorphics (e.g., Rapamycin, JAK inhibitors) suppress SASP without cell elimination. Epigenetic remodeling strategies include DNMT inhibitors (5-azacytidine), EZH2 inhibitors (Tazemetostat), HDAC inhibitors, BET inhibitors (JQ1), and CRISPR-dCas9-based precision epigenome editing. Cellular reprogramming approaches range from iPSC-derived notochordal cell transplantation to direct lineage reprogramming using transcription factor cocktails. The synergistic “clear-prime-seed” roadmap sequentially integrates these strategies: senescent cell clearance creates a permissive niche, epigenetic priming reactivates dormant progenitors, and *in situ* reprogramming or cell transplantation achieves functional tissue regeneration (Created by the Biorender).

Although this synergistic strategy remains at the proof-of-concept stage (Guo et al., 2022), it has already demonstrated potential in regenerative studies of other tissues (Fatima and Nawaz, 2017), charting a promising course toward curative intervention for IDD (Figure 4) (Table 2).

Operationalizing each phase requires specific refinements. Phase 1 “Clear”: precision depletion using subset-specific approaches (e.g., PROTACs against DPP4 or uPAR enriched on Fibro-NPCs, or sequential senolytic regimens that spare progenitors) (Winkler et al., 2016). Phase 2 “Prime”: a multi-pronged approach combining epigenetic de-repression (EZH2/HDAC inhibitors), microenvironmental dampening (BET inhibitors) and mechanosensory resetting (RhoA/ROCK inhibition) to create a pro-regenerative niche (Winkler et al., 2016; Kandarpa et al., 2019). Phase 3 “Seed”: *in situ* chemical/mRNA reprogramming of resident cells is preferred (Liu Y. et al., 2024); transplantation of hypoxia-preconditioned iPSC-progenitors in laminin-functionalized hydrogels should be reserved for advanced degeneration where endogenous substrate is depleted (He et al., 2021).

While the “clear, prime, then seed” roadmap is conceptually compelling, its clinical implementation presents significant challenges. The optimal timing and interval between interventions remain undefined: senolytic-induced transient inflammation may blunt epigenetic priming if initiated too early, while delays allow new senescent cells to accumulate. Drug-drug interactions also pose risks—for example, HDAC inhibitors upregulate anti-apoptotic proteins that could reduce subsequent senolytic efficacy. Finally, multiple intradiscal injections carry cumulative risks of discitis, nerve injury, or herniation. Preclinical studies must therefore optimize not just individual components but their integrated sequence and timing.

## 5 Challenges, controversies, and future directions

Despite the compelling conceptual framework positioning IDD as an epigenetically driven aging-related disease and the promising therapeutic strategies emerging from this paradigm, substantial hurdles remain on the path from bench to bedside. This section critically examines the theoretical and technical challenges confronting current research, dissects ongoing controversies within the field, and delineates potential avenues for future investigation based on these considerations.

### 5.1 Theoretical and conceptual challenges: Do we truly understand the IDD “clock”?

#### 5.1.1 The causality conundrum: Are epigenetic changes “drivers” or “passengers”?

A fundamental question remains unresolved: Do the epigenetic alterations observed during IDD progression—including aberrant DNA methylation and disrupted histone modification patterns—represent primary drivers of degeneration, or are they merely epiphenomena accompanying cellular senescence and matrix degradation? The preponderance of existing evidence derives from cross-sectional observations, which are inherently limited in establishing causal relationships (Gradisnik et al., 2025). For instance, the mechanistic link between SOX9 promoter hypermethylation and its subsequent transcriptional silencing has been relatively well established (Núñez-Carro et al., 2023). However, the initiating factors triggering this methylation event remain obscure. Is it mechanical stress, oxidative stress, or activation of upstream signaling pathways that serves as the primary instigator (Jin et al., 2022; Du et al., 2025; Xiang et al., 2023)? Addressing this question necessitates more refined temporal studies, employing approaches such as *in vitro* induced degeneration models that enable dynamic tracking of epigenetic mark evolution at multiple time points during degeneration, coupled with gain- and loss-of-function experiments for mechanistic validation.

To resolve causality, three experimental strategies are proposed (1) time-resolved multi-omics in animal models (e.g., WGBS and ATAC-seq at serial timepoints post-injury): to identify changes that precede senescence; (2) CRISPR-dCas9 epigenome editing to test whether locus-specific hypermethylation of SOX9 in healthy NPCs is sufficient to induce senescence, or whether demethylation in degenerated cells can restore function; and (3) *in vitro* induced degeneration models with dynamic ChIP-seq to establish the temporal hierarchy of chromatin state alterations.

#### 5.1.2 What constitutes the “youthful” blueprint? The knowledge gap in healthy reference states

If the objective of reprogramming is to “reset” degenerated cells to a healthy state, then a prerequisite must first be established: what precisely constitutes the complete epigenetic blueprint of healthy, youthful NP cells? Currently, our understanding of the epigenetic landscape of human intervertebral disc cells from healthy individuals remains remarkably limited. The majority of studies rely on control tissues obtained from cadaveric donors or patients undergoing scoliosis surgery—specimens that may themselves harbor subclinical degenerative changes or developmental abnormalities (Veronesi et al., 2026; Li Z. et al., 2022; Shi et al., 2023). The construction of a high-resolution, multi-age, degeneration-free epigenetic reference atlas of human intervertebral disc cells—encompassing DNA methylation, histone modifications, chromatin accessibility, and other dimensions—represents a foundational endeavor essential for all future precision epigenetic intervention studies. The application of spatial multi-omics technologies will prove particularly crucial, as these approaches can unveil the epigenetic heterogeneity among cells residing in different regions within the same intervertebral disc (Swahn et al., 2024).

#### 5.1.3 Etiological and molecular heterogeneity

IDD does not represent a single disease entity but rather encompasses substantial inter-individual variability in etiology, progression rate, and pain phenotype. Currently prevalent imaging-based assessments, such as the Pfirrmann grading system, inadequately capture the underlying molecular heterogeneity. A critical question emerges: do the operational mechanisms of the epigenetic “clock” differ across IDD cases driven by distinct etiologies? Furthermore, do qualitative differences exist between the epigenetic signatures of early- versus late-stage IDD?

IDD comprises at least four subtypes: mechanical (Piezo1/ROCK-driven, early histone modifications), metabolic (SIRT1/mTOR dysregulation, global hypomethylation), aging-associated (epigenetic clock acceleration, retrotransposon activation) and post-traumatic (focal inflammation, acute SASP). These subtypes likely exhibit distinct epigenetic dependencies—e.g., chromatin accessibility changes in mechanical IDD vs. DNA methylation remodeling in metabolic IDD. Matching therapies to subtypes will require composite stratification panels integrating imaging, clinical phenotype and molecular markers (serum SASP, cfDNA methylation), enabling personalized regimens such as Senomorphics for inflammatory SASP-dominant disease vs. combined Senolytic-EZH2 inhibitor therapy for accelerated epigenetic aging. The identification of epigenetic biomarkers associated with specific disease stages and subtypes will be essential for patient stratification and the development of personalized therapeutic strategies (Silwal et al., 2023; Samanta et al., 2023; Tong et al., 2022).

### 5.2 Technical and translational challenges: translating concepts into therapies

#### 5.2.1 Delivery barriers: traversing the AF to reach the NP

The intervertebral disc constitutes the largest avascular organ in the human body—an anatomical feature that simultaneously underlies its physiological characteristics and poses the most formidable obstacle to drug delivery. The dense, lamellar structure of the AF functions as a natural barrier, precluding systemically administered therapeutics from achieving effective concentrations within the NP (Frost et al., 2019). Consequently, local injection represents virtually the only viable administration route (Samanta et al., 2023). However, the elevated intradiscal pressure and rapid clearance following injection result in exceptionally short half-lives for single-dose administrations (Zhang J. et al., 2022). The development of advanced delivery systems capable of sustained, targeted, and controlled release has thus become an urgent priority. Current research directions encompass:

##### 5.2.1.1 Smart hydrogels

These materials undergo *in situ* gelation following injection and can be engineered to respond to microenvironmental signals, enabling temporally sequenced drug release (Malekmohammadi et al., 2021).

##### 5.2.1.2 Nanoparticles/microspheres

These carriers encapsulate drugs or nucleic acids, protecting them from enzymatic degradation while surface modifications can enhance cellular uptake efficiency (Mahapatro and Singh, 2011; Yetisgin et al., 2020).

##### 5.2.1.3 Extracellular vesicles

As naturally occurring nanoscale delivery vehicles, extracellular vesicles offer low immunogenicity and favorable tissue penetration capabilities, with the capacity to transport miRNAs, mRNAs, and even small molecule drugs (Herrmann et al., 2021; Kumar et al., 2024).

Equally important is biomechanical and spatiotemporal compatibility with the intervertebral disc environment. The injection process generates high shear forces, requiring shear-stable formulations; injectable nanostructured colloidal gels that mimic the shear-thinning and self-healing properties of native NP have been developed to maintain integrity during delivery (Wang Y. et al., 2021). Once injected, any hydrogel must match the swelling pressure and modulus of the native NP to avoid extrusion—an acknowledged failure mode of NP replacements, which can be mitigated by materials that expand *in situ* to resist migration (National Center for Biotechnology Information, 2026). Crucially, pathology-responsive sequential release can be achieved by coupling hydrogel degradation to disc-specific enzymatic activity. A dual-pathological cascade delivery system has been reported in which an ROS-responsive hydrogel first releases MMP13-sensitive apoptotic vesicles; elevated MMP13 in the degenerated disc then triggers secondary cargo liberation, providing closed-loop, pathology-responsive delivery from a single injection (Chen et al., 2025). Similarly, MMP-cleavable peptide crosslinkers have been used to engineer injectable hydrogels that undergo on-demand degradation in the MMP-rich degenerative disc microenvironment, enabling bioresponsive drug release (Feng G. et al., 2018). These proof-of-concept systems illustrate how material engineering can harness the biochemical and biomechanical features of the disc to achieve spatiotemporally controlled, multi-step therapy.

#### 5.2.2 Impact of the unique disc microenvironment on cell survival and function

Even when therapeutic agents or cells successfully reach the NP, they must contend with an exceptionally hostile microenvironment characterized by low oxygen tension, acidic pH, elevated osmotic pressure, nutrient deprivation, and dynamic mechanical loading (Guerrero et al., 2021). Within such an environment, a fundamental question arises: can exogenously implanted stem cells or reprogrammed cells survive, proliferate, and maintain their intended functional phenotype? Studies have demonstrated that stem cells cultured under conventional normoxic conditions undergo substantial cell death upon transplantation into the hypoxic disc environment due to metabolic maladaptation (Guerrero et al., 2021; Sang et al., 2025). Future regenerative strategies must therefore rigorously address this issue of “microenvironmental compatibility.” Potential solutions under investigation include:

##### 5.2.2.1 Preconditioning culture

*In vitro* cultivation of cells under conditions mimicking the disc microenvironment to promote adaptive responses prior to transplantation (Hu and Li, 2018; Ho et al., 2018; Jimoh et al., 2025).

##### 5.2.2.2 Co-delivery of cytoprotective agents

Administration of antioxidants, anti-apoptotic factors, or other protective molecules alongside cells or therapeutic compounds (Jimoh et al., 2025; Pirmoradi et al., 2023; Abbas et al., 2025).

##### 5.2.2.3 Material-mediated microenvironment modulation

Utilization of biomaterials that locally buffer pH, scavenge reactive oxygen species, or provide metabolic substrates, thereby creating a “protective micro-niche” for transplanted or resident cells (Jia et al., 2023; Zhang et al., 2025; Zhang J. et al., 2025).

Rather than solely a barrier, the distinctive physicochemical features of the disc microenvironment can be harnessed as endogenous triggers for controlled therapeutic activation. Hypoxia-responsive circuits: The NP is physiologically hypoxic, with HIF-1α serving as the master transcriptional regulator that activates target genes via hypoxia response elements (HREs) (Kim et al., 2021); the functionality of HRE motifs in NP cells has been directly demonstrated through HIF-dependent regulation of endogenous genes (Skubutyte et al., 2010). Placing reprogramming factors under HRE control would, in principle, restrict their translation to the hypoxic NP environment. pH-responsive delivery: The acidic pH (6.2–6.8) characteristic of degenerated discs can be exploited for lesion-specific drug release. pH-responsive hydrogels that deliver therapeutic agents selectively in the acidic disc environment have been shown to improve disc height and alleviate pain in rat models (Wang et al., 2024), while dual ROS/pH-responsive hydrogel-nanoparticle systems have demonstrated efficacy across multiple animal models (Zhong et al., 2026). Metabolic pre-adaptation: Hypoxic preconditioning enhances the survival and migration of transplanted mesenchymal stem cells, retarding disc degeneration *in vivo* (Vadalà et al., 2019; Wang W. et al., 2018; Liang et al., 2012). The concept of mTOR-mediated metabolic pre-adaptation is supported by evidence that quiescence preconditioning driven by serum deprivation—operating through mTOR pathway inhibition and autophagy activation—enhances stromal cell survival under severe ischemia (Chen Q. et al., 2023). Transient rapamycin pre-treatment to induce autophagy competence before nutrient-restricted culture may therefore enhance the metabolic resilience of therapeutic cells, though integration with NP-specific pre-adaptation protocols has not yet been experimentally tested.

#### 5.2.3 Model limitations: the translational gap from animals to humans

The preponderance of current evidence derives from *in vitro* cellular experiments and rodent animal models. However, the intervertebral discs of mice, rats, and even rabbits exhibit substantial differences from humans in terms of anatomical structure, cellular composition, mechanical loading, and degenerative patterns (Alini et al., 2007; Daly et al., 2016). Notably, rodents retain notochordal cells throughout their lifespan, whereas in humans, these cells are replaced by chondrocyte-like cells following adolescence—a fundamental difference that renders degenerative mechanisms distinct between species (Daly et al., 2016; Risbud et al., 2010). Consequently, positive results obtained from rodent models frequently fail to recapitulate in large animal studies or human applications. The development of human-relevant model systems that more closely mirror physiological and pathological states has thus become imperative:

##### 5.2.3.1 Disc organoids

Utilizing human iPSCs or multipotent stem cells, three-dimensional culture systems enable self-assembly of “miniature intervertebral discs” incorporating NP, AF, and CEP structures, providing valuable platforms for drug screening and mechanistic investigations (Yang et al., 2023; Scalise et al., 2021; Cai et al., 2026).

##### 5.2.3.2 Organs-on-chips

Microfluidic devices engineered to recapitulate functional units of the intervertebral disc permit precise control over oxygen gradients, nutrient supply, and mechanical loading, thereby simulating the complex *in vivo* microenvironment (Wang et al., 2026; Fritschen et al., 2022).

#### 5.2.4 Clinical trial design hurdles

Translational trials for IDD face unique challenges: (1) patient stratification—molecular biomarkers (serum SASP profiles, cfDNA methylation) must complement MRI to select appropriate subtypes; (2) pharmacodynamic endpoints—target engagement should be directly verified in disc biopsies (e.g., H3K27me3 reduction by ChIP-qPCR after EZH2 inhibitor treatment); and (3) ethical constraints—the relatively young IDD population demands higher safety margins than oncology, necessitating local delivery and extended long-term follow-up.

### 5.3 Biological and safety concerns: What are the costs of intervening in aging?

#### 5.3.1 Multi-level safety challenges

1. Epigenetic drugs: Pan-inhibitors risk reactivating endogenous retroviruses, demethylating oncogene promoters, and disrupting imprinting. Mitigation includes developing subtype-selective inhibitors (e.g., HDAC9-specific) and CRISPR-dCas9 editing for locus-specific modification, though long-term immunogenicity and off-target binding require large-animal validation (Soto-Palm et al., 2022; Dai et al., 2021).
2. Senolytics: Indiscriminate elimination may deplete beneficial progenitor cells (e.g., Tie2+ NPPCs) or transient senescence required for wound healing. Solutions include prodrugs activated by SA-β-gal, ADCs against senescence-specific antigens (DPP4, uPAR), or preferential use of Senomorphics (Power et al., 2023; Zhang L. et al., 2022).
3. Reprogramming: iPSC teratoma risk and incomplete reprogramming-associated intermediates may drive tumorigenesis (Aguirre et al., 2023; Huyghe et al., 2023; Cerneckis et al., 2024). Safer options include non-integrating vectors (modified mRNA, Sendai virus), elimination of c-MYC from cocktails, and chemical reprogramming. An inducible suicide gene (e.g., iCasp9) provides an additional safety switch (Steinle et al., 2016; Warren et al., 2012).

#### 5.3.2 Long-term consequences of epigenetic editing

Sustained dCas9-effector expression may accumulate off-target modifications over years in the disc niche. AAV vectors face limited cargo capacity (∼4.7 kb), necessitating dual-AAV systems or smaller Cas orthologs. Non-viral alternatives (LNPs, extracellular vesicles) offer transient expression and lower immunogenicity but poorer *in vivo* delivery efficiency to the avascular disc. Solving this trade-off is essential for safe translation.

#### 5.3.3 Ethical and societal considerations

As the concept of “reversing aging” transitions from science fiction toward clinical reality, attendant ethical questions emerge. For instance, the clinical application of autologous iPSC therapy for IDD needs to meet the regulatory requirements of cell therapy products, and the off-target effect of *in vivo* CRISPR epigenetic editing also faces strict ethical review.

If we can “reset” the aging clock of the intervertebral disc, does this imply attempts to reverse the biological age of the entire organism? What impact might such localized interventions exert on systemic aging processes? Could the high costs of regenerative therapies exacerbate existing healthcare disparities? Although these questions extend beyond the primary scope of this review, their appropriate acknowledgment serves to contextualize the broader academic and humanistic implications of this research Frontier.

### 5.4 Future directions: the path toward clinical translation

#### 5.4.1 A biomarker framework for precision IDD therapy

##### 5.4.1.1 Diagnostic/stratification markers

A composite panel of serum SASP proteins (IL-6, CXCL10, MMP-3) (Weber et al., 2016; Yang et al., 2022; Gao et al., 2025), disc-derived cfDNA methylation signatures at key loci (SOX9, ACAN) (Milheiro et al., 2025; Hou et al., 2026; Shetty et al., 2026), and quantitative MRI (T1ρ, MTR) can differentiate molecular subtypes (Antoniou et al., 2013; Pandit et al., 2016).

##### 5.4.1.2 Pharmacodynamic (PD) markers

Minimally invasive disc biopsies should directly confirm target engagement—e.g., ChIP-qPCR for H3K27me3 at FOXF1 after EZH2 inhibition, or scRNA-seq to verify NPC identity gene de-repression post-therapy (Yao et al., 2024; Egan et al., 2016; Li Z. et al., 2022; Jiang et al., 2019).

##### 5.4.1.3 Imaging surrogate endpoints

MTR is particularly sensitive to macromolecular matrix restoration and may serve as an early efficacy readout complementing patient-reported outcomes (Antoniou et al., 2013; Pandit et al., 2016).

#### 5.4.2 Mapping the epigenetic aging atlas of the human intervertebral disc

Leveraging single-cell multi-omics technologies integrated with spatial transcriptomics, the construction of a comprehensive cellular atlas of the human intervertebral disc encompassing diverse ages, degenerative stages, and etiologies represents a foundational priority. The objectives are threefold: to identify key cellular subpopulations driving IDD and their characteristic epigenetic alterations; to establish an “epigenetic clock” for IDD that enables precise prediction of disease progression; and to discover novel therapeutic targets and diagnostic biomarkers (Feng et al., 2023; Stirnimann et al., 2025; Chen C. et al., 2023; Wang et al., 2025).

#### 5.4.3 Development of microenvironment-responsive “smart” delivery platforms

Research focus should converge on injectable, biodegradable smart hydrogels and engineered extracellular vesicles capable of achieving the following functionalities: temporal multi-drug release—sequenced delivery of senolytics for “clearance,” followed by epigenetic modifiers for “priming,” and finally reprogramming factors for “seeding” (Nguyen, 2023; Xie et al., 2022; Hashemi et al., 2024); closed-loop regulation—materials engineered to sense local microenvironmental signals indicative of inflammation or senescence and autonomously adjust drug release kinetics accordingly (Liu J. et al., 2023; Chen Y. et al., 2023); and physicochemical synergy—integration of mechanical support provided by biomaterials with biological modulation via therapeutic agents to achieve dual restoration of structure and function (Choi et al., 2024; Pishavar et al., 2021).

#### 5.4.4 Advancing CRISPR-dCas9 epigenetic editing through preclinical studies

CRISPR-dCas9-based epigenetic editing represents the ultimate embodiment of “precision medicine.” Future investigations should prioritize: screening of safe and efficient *in vivo* delivery vectors; and validation of efficacy and long-term safety in large animal models through precise activation of SOX9 or silencing of MMP13 as therapeutic interventions for IDD (Jia et al., 2024; Meng Y. et al., 2025; Cai et al., 2023; Azeez et al., 2024).

#### 5.4.5 Initiation of early-phase clinical trials with biomarker-based stratification

**FIGURE 5:**
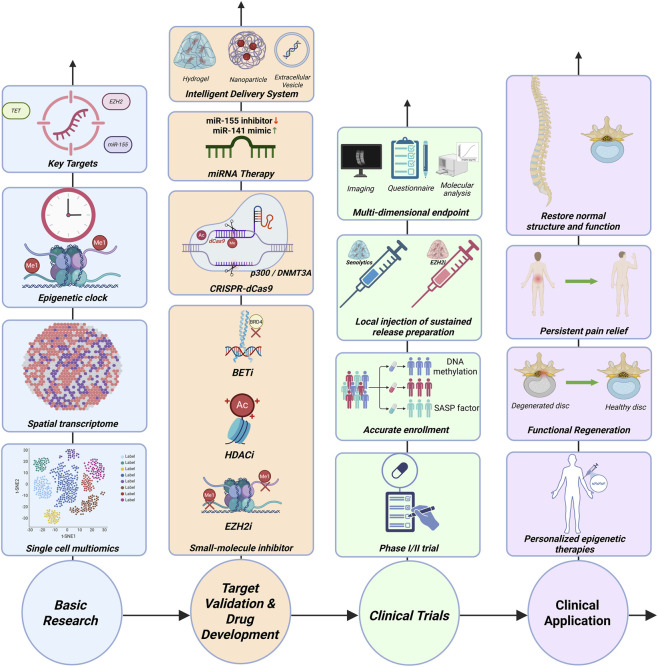
Roadmap for epigenetic-based therapies in IDD: from discovery to clinic. The translational pathway for epigenetic therapies in IDD progresses through five integrated phases: (1) construction of a multi-age, multi-stage human disc epigenetic reference atlas using single-cell multi-omics and spatial transcriptomics; (2) establishment of a validated IDD-specific epigenetic clock for precise disease staging and progression prediction; (3) development of microenvironment-responsive “smart” delivery platforms enabling temporally sequenced drug release; (4) preclinical validation of CRISPR-dCas9 epigenetic editing in large animal models with long-term safety monitoring; and (5) initiation of early-phase clinical trials incorporating biomarker-based patient stratification and pharmacodynamic target-engagement endpoints (Created by the Biorender).

Prior to embarking on clinical trials, stringent inclusion and exclusion criteria must be established. Future early-phase trials should focus on: target populations—patients with early-to-mid stage IDD presenting definitive pain symptoms refractory to conservative management; interventional approaches—agents with preliminary safety validation, such as locally injected low-dose senolytics or EZH2 inhibitors encapsulated within sustained-release hydrogels; and endpoint measures—beyond conventional imaging and patient-reported outcomes, incorporation of molecular-level mechanistic validation to confirm that therapeutic interventions indeed engage the intended biological targets (Figure 5).

### 5.5 Translational priority framework

To guide resource allocation and clinical development, we propose a tiered approach based on preclinical evidence, clinical experience, and technical feasibility:

#### 5.5.1 Immediate priority

Local repurposed senolytics/senomorphicsDasatinib, quercetin, and rapamycin have established safety profiles and robust preclinical IDD data. Early trials should evaluate sustained-release hydrogel formulations in early-to-mid stage IDD (Pfirrmann II-III).

#### 5.5.2 Near-term priority

Small molecule epigenetic modifiersEZH2 and BET inhibitors show strong preclinical efficacy and are in late-stage cancer development. Initial trials should prioritize pharmacodynamic endpoints (H3K27me3 levels, SOX9 expression) in disc biopsies.

#### 5.5.3 Mid-term priority

RNA-based therapeuticsmiRNA mimics/inhibitors offer improved specificity and benefit from established regulatory pathways. Advances in lipid nanoparticle and extracellular vesicle delivery are addressing historical stability and uptake challenges.

#### 5.5.4 Long-term priority

CRISPR-dCas9 editing and *in vivo* reprogramming These curative approaches require resolution of delivery, off-target, and long-term safety issues. Large animal studies in sheep or pigs are critical for validating human-relevant efficacy.

This framework is dynamic and will evolve with technological breakthroughs, particularly in delivery systems that could accelerate RNA and CRISPR-based approaches.

## 6 Conclusion

IDD is not a passive mechanical wear process, but a programmed cellular degeneration process driven by epigenetic clock dysregulation, which is characterized by the loss of NPC identity and the establishment of a self-amplifying senescence-associated microenvironment. This review systematically resolves the cascade mechanism between epigenetic dysregulation, cellular senescence and microenvironmental deterioration in IDD, distinguishes the core controversies in the field, and proposes a sequential “clear, prime, then seed” synergistic regeneration strategy for the first time, which breaks through the cognitive limitation of traditional symptomatic treatment for IDD. In the future, the construction of a standardized IDD epigenetic clock, the development of cell-specific targeted delivery systems, and the verification of the synergistic regeneration strategy in large animal models will be the core directions to promote the clinical translation of IDD epigenetic therapy.

## References

[B1] Abbas S. E. M. Maged G. Wang H. Lotfy A. (2025). Mesenchymal stem/stromal cells microencapsulation for cell therapy. Cells 14 (3), 149. 10.3390/cells14030149 39936941 PMC11817150

[B2] Acosta J. C. Banito A. Wuestefeld T. Georgilis A. Janich P. Morton J. P. (2013). A complex secretory program orchestrated by the inflammasome controls paracrine senescence. Nat. Cell Biol. 15 (8), 978–990. 10.1038/ncb2784 23770676 PMC3732483

[B3] Aguirre M. Escobar M. Forero Amézquita S. Cubillos D. Rincón C. Vanegas P. (2023). Application of the Yamanaka transcription factors Oct4, Sox2, Klf4, and c-Myc from the laboratory to the clinic. Genes 14 (9), 1697. 10.3390/genes14091697 37761837 PMC10531188

[B4] Alini M. Eisenstein S. M. Ito K. Little C. Kettler A. A. Masuda K. (2007). Are animal models useful for studying human disc disorders/degeneration? Eur. Spine J. 17 (1), 2–19. 10.1007/s00586-007-0414-y 17632738 PMC2365516

[B5] Alqahtani S. Alqahtani T. Venkatesan K. Sivadasan D. Ahmed R. Sirag N. (2025). SASP modulation for cellular rejuvenation and tissue homeostasis: therapeutic strategies and molecular insights. Cells 14 (8), 608. 10.3390/cells14080608 40277933 PMC12025513

[B6] Antoniou J. Epure L. M. Michalek A. J. Grant M. P. Iatridis J. C. Mwale F. (2013). Analysis of quantitative magnetic resonance imaging and biomechanical parameters on human discs with different grades of degeneration. J. Magnetic Resonance Imaging 38 (6), 1402–1414. 10.1002/jmri.24120 23633131 PMC3735720

[B7] Ashruf O. S. Ansari M. Y. (2022). Natural compounds: potential therapeutics for the inhibition of cartilage matrix degradation in osteoarthritis. Life 13 (1), 102. 10.3390/life13010102 36676051 PMC9866583

[B8] Aunan J. R. Cho W. C. Søreide K. (2017). The biology of aging and cancer: a brief overview of shared and divergent molecular hallmarks. Aging Dis. 8 (5), 628–642. 10.14336/ad.2017.0103 28966806 PMC5614326

[B9] Azeez S. S. Hamad R. S. Hamad B. K. Shekha M. S. Bergsten P. (2024). Advances in CRISPR-Cas technology and its applications: revolutionising precision medicine. Front. Genome. 6, 1509924. 10.3389/fgeed.2024.1509924 PMC11669675 39726634

[B10] Bao C. Zhu W. Bao T. Hou Y. Wu T. Huang J. (2025). The application of epigenetic clocks in degenerative musculoskeletal diseases: a systematic review. Osteoarthr. Cartil. 33 (9), 1052–1065. 10.1016/j.joca.2025.05.003 40409455

[B11] Cai R. Lv R. Shi X. Yang G. Jin J. (2023). CRISPR/dCas9 tools: epigenetic mechanism and application in gene transcriptional regulation. Int. J. Mol. Sci. 24 (19), 14865. 10.3390/ijms241914865 37834313 PMC10573330

[B12] Cai Lu Ru B. Ren H. Cai F. Zeng L. Yang J. (2026). Gas therapy: an innovative application for intervertebral disc degeneration. Med. Gas Res. 16 (3), 293–302. 10.4103/mgr.MEDGASRES-D-25-00191 41496306 PMC12935121

[B13] Cao L. Li K. Li Q. Tong Q. Wang Y. Huang L. (2025). The controversial role of senescence-associated secretory phenotype (SASP) in cancer therapy. Mol. Cancer 24 (1), 283. 10.1186/s12943-025-02475-8 41204284 PMC12595836

[B14] Cerneckis J. Cai H. Shi Y. (2024). Induced pluripotent stem cells (iPSCs): molecular mechanisms of induction and applications. Signal Transduct. Target. Ther. 9 (1), 112. 10.1038/s41392-024-01809-0 38670977 PMC11053163

[B15] Chen C. Wang J. Pan D. Wang X. Xu Y. Yan J. (2023). Applications of multi‐omics analysis in human diseases. MedComm 4.4, e315. 10.1002/mco2.315 37533767 PMC10390758

[B16] Chen Y. Wang X. Tao S. Wang Q. Ma P. Q. Li Z. B. (2023). Research advances in smart responsive-hydrogel dressings with potential clinical diabetic wound healing properties. Mil. Med. Res. 10 (1), 37. 10.1186/s40779-023-00473-9 37608335 PMC10463485

[B17] Chen Q. Yang Q. Pan C. Ding R. Wu T. Cao J. (2023). Quiescence preconditioned nucleus pulposus stem cells alleviate intervertebral disc degeneration by enhancing cell survival via adaptive metabolism pattern in rats. Front. Bioengineer. Biotechnol. 11, 1073238. 10.3389/fbioe.2023.1073238 PMC9950514 36845177

[B18] Chen W. Zhao T. Ren Y. Huang W. Xia J. Hu Z. (2025). Dual-pathological cascade delivery of apoptotic vesicles for targeted therapy in intervertebral disc degeneration. Mater. Today. Bio 34, 102200. 10.1016/j.mtbio.2025.102200 40893365 PMC12391270

[B19] Cheng P. Wei H. Chen H. Wang Z. Mao P. Zhang H. (2023). DNMT3a‐mediated methylation of PPARγ promote intervertebral disc degeneration by regulating the NF‐κB pathway. J. Cell. Mol. Med. 28 (2), e18048. 10.1111/jcmm.18048 37986543 PMC10826446

[B20] Cheng Z. Gao H. Shi P. Zhang A. Chen X. Chen Y. (2025). Chaperone-mediated autophagy directs a dual mechanism to balance premature senescence and senolysis to prevent intervertebral disc degeneration. Bone Res. 13 (1), 62. 10.1038/s41413-025-00441-0 40506462 PMC12162876

[B21] Cheng P. Li H. Chen H. W. Wang Z. Q. Li P. w. Zhang H. H. (2025). 5-Azacytidine inhibits endoplasmic reticulum stress and apoptosis of nucleus pulposus cells by preserving PPARγ via promoter demethylation. In Vitro Cell. Dev. Biol. Anim. 61 (3), 288–297. 10.1007/s11626-025-01021-6 40102314

[B22] Choi H. Choi W. S. Jeong J. O. (2024). A review of advanced hydrogel applications for tissue engineering and drug delivery systems as biomaterials. Gels 10 (11), 693. 10.3390/gels10110693 39590049 PMC11594258

[B23] Chung H. Y. Kim D. H. Lee E. K. Chung K. W. Chung S. Lee B. (2018). Redefining chronic inflammation in aging and age-related diseases: proposal of the senoinflammation concept. Aging Dis. 10 (2), 367–382. 10.14336/ad.2018.0324 31011483 PMC6457053

[B24] Coppé J. P. Desprez P. Y. Krtolica A. Campisi J. (2010). The senescence-associated secretory phenotype: the dark side of tumor suppression. Annu. Rev. Pathol. 5, 99–118. 10.1146/annurev-pathol-121808-102144 20078217 PMC4166495

[B25] Dai E. Zhu Z. Wahed S. Qu Z. Storkus W. J. Guo Z. S. (2021). Epigenetic modulation of antitumor immunity for improved cancer immunotherapy. Mol. Cancer 20, 1. 10.1186/s12943-021-01464-x 34930302 PMC8691037

[B26] Daly C. Ghosh P. Jenkin G. Oehme D. Goldschlager T. (2016). A review of animal models of intervertebral disc degeneration: pathophysiology, regeneration, and translation to the clinic. BioMed Res. Int. 2016, 1–14. 10.1155/2016/5952165 27314030 PMC4893450

[B27] Davan-Wetton C. S. A. Pessolano E. Perretti M. Montero-Melendez T. (2021). Senescence under appraisal: hopes and challenges revisited. Cell. Mol. Life Sci. 78 (7), 3333–3354. 10.1007/s00018-020-03746-x 33439271 PMC8038995

[B28] Dekker J. Mirny L. A. (2024). The chromosome folding problem and how cells solve it. Cell 187 (23), 6424–6450. 10.1016/j.cell.2024.10.026 39547207 PMC11569382

[B29] Deng P. Yu Y. Hong C. Wang C. Y. (2020). Growth differentiation factor 6, a repressive target of EZH2, promotes the commitment of human embryonic stem cells to mesenchymal stem cells. Bone Res. 8 (1), 39. 10.1038/s41413-020-00116-y 33298857 PMC7672114

[B30] Dowdell J. Erwin M. Choma T. Vaccaro A. Iatridis J. Cho S. K. (2017). Intervertebral disk degeneration and repair. Neurosurgery 80 (3S), S46–S54. 10.1093/neuros/nyw078 28350945 PMC5585783

[B31] Du Q. Xue J. Fan D. Chen J. Zhong G. Chen J. (2025). CircZNF418 alleviates oxidative stress-induced cartilage endplate degeneration by stabilizing Sox9 through HuR. J. Molecular Histology 56 (4), 242. 10.1007/s10735-025-10528-x 40742571

[B32] Egan B. Yuan C. C. Craske M. L. Labhart P. Guler G. D. Arnott D. (2016). An alternative approach to ChIP-Seq normalization enables detection of genome-wide changes in histone H3 lysine 27 trimethylation upon EZH2 inhibition. PLoS One 11 (11), e0166438. 10.1371/journal.pone.0166438 27875550 PMC5119738

[B33] Fang J. Hsueh Y. Y. Soto J. Sun W. Wang J. Gu Z. (2020). Engineering biomaterials with micro/nanotechnologies for cell reprogramming. ACS Nano 14 (2), 1296–1318. 10.1021/acsnano.9b04837 32011856 PMC10067273

[B34] Fatima F. Nawaz M. (2017). Vesiculated long non-coding RNAs: offshore packages deciphering trans-regulation between cells, cancer progression and resistance to therapies. Non-Coding RNA 3 (1), 10. 10.3390/ncrna3010010 29657282 PMC5831998

[B35] Feng C. Yang M. Lan M. Liu C. Zhang Y. Huang B. (2017). ROS: crucial intermediators in the pathogenesis of intervertebral disc degeneration. Oxidative Med. Cell. Longevity 2017, 5601593. 10.1155/2017/5601593 28392887 PMC5368368

[B36] Feng C. Yang M. Zhang Y. Lan M. Huang B. Liu H. (2018). Cyclic mechanical tension reinforces DNA damage and activates the p53-p21-Rb pathway to induce premature senescence of nucleus pulposus cells. Int. J. Mol. Med. 41 (6), 3316–3326. 10.3892/ijmm.2018.3522 29512682 PMC5881642

[B37] Feng G. Zha Z. Huang Y. Li J. Wang Y. Ke W. (2018). Sustained and bioresponsive two-stage delivery of therapeutic miRNA via polyplex micelle-loaded injectable hydrogels for inhibition of intervertebral disc fibrosis. Adv. Healthc. Mater. 7 (21), e1800623. 10.1002/adhm.201800623 30296017

[B38] Feng S. Li J. Tian J. Lu S. Zhao Y. (2023). Application of single-cell and spatial omics in musculoskeletal disorder research. Int. J. Mol. Sci. 24 (3), 2271. 10.3390/ijms24032271 36768592 PMC9917071

[B39] Feng T. Xie F. Lee L. M. Lin Z. Tu Y. Lyu Y. (2025). Cellular senescence in cancer: from mechanism paradoxes to precision therapeutics. Mol. Cancer 24 (1), 213. 10.1186/s12943-025-02419-2 40781676 PMC12333312

[B40] Fritschen A. Bell A. K. Königstein I. Stühn L. Stark R. W. Blaeser A. (2022). Investigation and comparison of resin materials in transparent DLP-printing for application in cell culture and organs-on-a-chip. Biomater. Sci. 10 (8), 1981–1994. 10.1039/d1bm01794b 35262097

[B41] Frost B. A. Camarero-Espinosa S. Foster E. J. (2019). Materials for the spine: anatomy, problems, and solutions. Materials 12 (2), 253. 10.3390/ma12020253 30646556 PMC6356370

[B42] Gan Y. He J. Zhu J. Xu Z. Wang Z. Yan J. (2021). Spatially defined single-cell transcriptional profiling characterizes diverse chondrocyte subtypes and nucleus pulposus progenitors in human intervertebral discs. Bone Res. 9 (1), 37. 10.1038/s41413-021-00163-z 34400611 PMC8368097

[B43] Gao Y. Ma X. Shi Z. Zhu M. Yang Z. Tao Z. (2025). MMP expression and its clinical significance in intervertebral disc destruction of spinal tuberculosis, Brucellar spondylitis, and pyogenic spondylitis. J. Orthopaedic Surg. Res. 20 (1), 208. 10.1186/s13018-025-05622-5 40016774 PMC11866808

[B44] GBD 2021 US Burden of Disease Collaborators (2024). The burden of diseases, injuries, and risk factors by state in the USA, 1990-2021: a systematic analysis for the Global Burden of Disease Study 2021. Lancet (London, England) 404, 2314–2340. 10.1016/S0140-6736(24)01446-6 39645376 PMC11694014

[B45] Gradisnik L. Kocivnik N. Maver U. Velnar T. (2025). Degenerative disease of intervertebral disc: a narrative review of pathogenesis, clinical implications and therapies. Bioengineering 13 (1), 40. 10.3390/bioengineering13010040 41595972 PMC12837267

[B46] Guerrero J. Häckel S. Croft A. Hoppe S. Albers C. Gantenbein B. (2021). The nucleus pulposus microenvironment in the intervertebral disc: the fountain of youth? Eur. Cells Mater. 41, 707–738. 10.22203/ecm.v041a46 34128534

[B47] Guo H. Y. Guo M. K. Wan Z. Y. Song F. Wang H. Q. (2020). Emerging evidence on noncoding-RNA regulatory machinery in intervertebral disc degeneration: a narrative review. Arthritis Res. Ther. 22 (1), 270. 10.1186/s13075-020-02353-2 33198793 PMC7667735

[B48] Guo J. Huang X. Dou L. Yan M. Shen T. Tang W. (2022). Aging and aging-related diseases: from molecular mechanisms to interventions and treatments. Signal Transduct. Target. Ther. 7 (1), 391. 10.1038/s41392-022-01251-0 36522308 PMC9755275

[B49] Guo S. Wang C. Xiao C. Gu Q. Long L. Wang X. (2023). Role of the mechanosensitive piezo1 channel in intervertebral disc degeneration. Clin. Physiol. Funct. Imaging 43 (2), 59–70. 10.1111/cpf.12798 36400723

[B50] Hamann A. Nguyen A. Pannier A. K. (2019). Nucleic acid delivery to mesenchymal stem cells: a review of nonviral methods and applications. J. Biol. Eng. 13 (1), 7. 10.1186/s13036-019-0140-0 30675180 PMC6339289

[B51] Hashemi A. Ezati M. Nasr M. P. Zumberg I. Provaznik V. (2024). Extracellular vesicles and hydrogels: an innovative approach to tissue regeneration. ACS Omega 9 (6), 6184–6218. 10.1021/acsomega.3c08280 38371801 PMC10870307

[B52] He X.-Y. Zhao C. Xu H. Chen K. Bian B. Gong Y. (2021). Synaptic repair and vision restoration in advanced degenerating eyes by transplantation of retinal progenitor cells. Stem Cell Rep. 16 (7), 1805–1817. 10.1016/j.stemcr.2021.06.002 PMC8282465 34214489

[B53] Hechavarria M. E. Richard S. A. (2022). Edifying the focal factors influencing mesenchymal stem cells by the microenvironment of intervertebral disc degeneration in low back pain. Pain Res. Manag. 2022, 1–12. 10.1155/2022/6235400 PMC8977320 35386857

[B54] Herrmann I. K. Wood M. J. A. Fuhrmann G. (2021). Extracellular vesicles as a next-generation drug delivery platform. Nat. Nanotechnol. 16 (7), 748–759. 10.1038/s41565-021-00931-2 34211166

[B55] Ho S. S. Hung B. P. Heyrani N. Lee M. A. Leach J. K. (2018). Hypoxic preconditioning of mesenchymal stem cells with subsequent spheroid formation accelerates repair of segmental bone defects. Stem Cells 36 (9), 1393–1403. 10.1002/stem.2853 29968952 PMC6125201

[B56] Holjencin C. E. Jakymiw A. (2022). MicroRNAs and their big therapeutic impacts: delivery strategies for cancer intervention. Cells 11 (15), 2332. 10.3390/cells11152332 35954176 PMC9367537

[B57] Hong J. Li S. Markova D. Z. Liang A. Kepler C. K. Huang Y. (2020). Bromodomain-containing protein 4 inhibition alleviates matrix degradation by enhancing autophagy and suppressing NLRP3 inflammasome activity in NP cells. J. Cell. Physiol. 235 (7-8), 5736–5749. 10.1002/jcp.29508 31975410

[B58] Horvath S. Raj K. (2018). DNA methylation-based biomarkers and the epigenetic clock theory of ageing. Nat. Rev. Genet. 19 (6), 371–384. 10.1038/s41576-018-0004-3 29643443

[B59] Horváth E. Sólyom Á. Székely J. Nagy E. E. Popoviciu H. (2023). Inflammatory and metabolic signaling interfaces of the hypertrophic and senescent chondrocyte phenotypes associated with osteoarthritis. Int. J. Mol. Sci. 24 (22), 16468. 10.3390/ijms242216468 38003658 PMC10671750

[B60] Hosokawa N. Hara T. Kaizuka T. Kishi C. Takamura A. Miura Y. (2009). Nutrient-dependent mTORC1 association with the ULK1-Atg13-FIP200 complex required for autophagy. Mol. Biol. Cell 20 (7), 1981–1991. 10.1091/mbc.e08-12-1248 19211835 PMC2663915

[B61] Hou Y. Liu L. Guo Y. Shi J. (2026). Epigenetic crossroads in intervertebral disc degeneration: unlocking novel therapeutic avenues. Mol. Med. Rep. 33 (4), 1–12. 10.3892/mmr.2026.13823 41685572 PMC12930325

[B62] Hu C. Li L. (2018). Preconditioning influences mesenchymal stem cell properties in vitro and in vivo. J. Cell. Mol. Med. 22 (3), 1428–1442. 10.1111/jcmm.13492 29392844 PMC5824372

[B63] Hu X. Y. Cao D. Z. Wang Z. H. (2021a). Metformin alleviates intervertebral disc degeneration by upregulating MMP-1 expression via the KDM6A/SOX9/miR-202-3p/MMP-1 signaling pathway. Arch. Med. Sci. 22 (1), 496–505. 10.5114/aoms/117427 42110643 PMC13154808

[B64] Hu X. li J. Fu M. Zhao X. Wang W. (2021b). The JAK/STAT signaling pathway: from bench to clinic. Signal Transduct. Target. Ther. 6 (1), 402. 10.1038/s41392-021-00791-1 34824210 PMC8617206

[B65] Huyghe A. Trajkova A. Lavial F. (2023). Cellular plasticity in reprogramming, rejuvenation and tumorigenesis: a pioneer TF perspective. Trends Cell Biol. 34 (3), 255–267. 10.1016/j.tcb.2023.07.013 37648593

[B66] Ito M. Yurube T. Kakutani K. Maeno K. Takada T. Terashima Y. (2017). Selective interference of mTORC1/RAPTOR protects against human disc cellular apoptosis, senescence, and extracellular matrix catabolism with Akt and autophagy induction. Osteoarthr. Cartilage 25 (12), 2134–2146. 10.1016/j.joca.2017.08.019 28888905

[B67] Jeon O. H. Kim C. Laberge R. M. Demaria M. Rathod S. Vasserot A. P. (2017). Local clearance of senescent cells attenuates the development of post-traumatic osteoarthritis and creates a pro-regenerative environment. Nat. Med. 23 (6), 775–781. 10.1038/nm.4324 28436958 PMC5785239

[B68] Ji S. Xiong M. Chen H. Liu Y. Zhou L. Hong Y. (2023). Cellular rejuvenation: molecular mechanisms and potential therapeutic interventions for diseases. Signal Transduct. Target. Ther. 8 (1), 116. 10.1038/s41392-023-01343-5 36918530 PMC10015098

[B69] Jia X. Dou Z. Zhang Y. Li F. Xing B. Hu Z. (2023). Smart responsive and controlled-release hydrogels for chronic wound treatment. Pharmaceutics 15 (12), 2735. 10.3390/pharmaceutics15122735 38140076 PMC10747460

[B70] Jia S. Liang R. Chen J. Liao S. Lin J. Li W. (2024). Emerging technology has a brilliant future: the CRISPR-Cas system for senescence, inflammation, and cartilage repair in osteoarthritis. Cell. Mol. Biol. Lett. 29 (1), 64. 10.1186/s11658-024-00581-x 38698311 PMC11067114

[B71] Jiang C. Guo Q. Jin Y. Xu J. J. Sun Z. M. Zhu D. C. (2019). Inhibition of EZH2 ameliorates cartilage endplate degeneration and attenuates the progression of intervertebral disc degeneration via demethylation of Sox-9. EBioMedicine 48, 619–629. 10.1016/j.ebiom.2019.10.006 31631036 PMC6838408

[B72] Jimoh O. O. Ajuwon T. Okonkwo S. S. Awoyemi R. F. Olaosebikan I. Olahanmi O. A. (2025). PLGA nanoparticles in otoprotection and inner ear regeneration: a new frontier in nanomedicine for hearing disorders. RSC Adv. 16 (1), 76–106. 10.1039/d5ra06007a 41438770 PMC12721246

[B73] Jing L. Francisco A. T. Chakraborty S. Leong K. W. Setton L. A. Chen J. (2014). Differentiation of human-induced pluripotent stem cells (iPSCs) on Laminin-functionalized PEG hydrogels under nucleus pulposus-like culture conditions. Glob. Spine J. 4 (Suppl l), 0034-1376571-s-0034-1376571. 10.1055/s-0034-1376571

[B74] Johnson Z. I. Schoepflin Z. R. Choi H. Shapiro I. M. Risbud M. V. (2015). Disc in flames: roles of TNF-α and IL-1β in intervertebral disc degeneration. Eur. Cells Mater. 30, 104–117. 10.22203/ecm.v030a08 26388614 PMC4751407

[B75] Kandarpa M. Peterson L. F. Potu H. Liu Y.-H. Polk A. Wang S. (2019). Superior pre-clinical efficacy of novel protac based BET degrader in a large acute myeloid leukemia cohort. Blood 134 (Suppl_1), 3936. 10.1182/blood-2019-131759

[B76] Kang L. Zhang H. Jia C. Zhang R. Shen C. (2023). Epigenetic modifications of inflammation in intervertebral disc degeneration. Ageing Res. Rev. 87, 101902. 10.1016/j.arr.2023.101902 36871778

[B77] Kim J. Kundu M. Viollet B. Guan K. L. (2011). AMPK and mTOR regulate autophagy through direct phosphorylation of Ulk1. Nat. Cell Biology 13 (2), 132–141. 10.1038/ncb2152 21258367 PMC3987946

[B78] Kim J. W. Jeon N. Shin D. E. Lee S. Y. Kim M. Han D. H. (2021). Regeneration in spinal disease: therapeutic role of hypoxia-inducible Factor-1 alpha in regeneration of degenerative intervertebral disc. Int. J. Mol. Sci. 22 (10), 5281. 10.3390/ijms22105281 34067899 PMC8155933

[B79] Kim H. Lebeau B. Papadopoli D. Jovanovic P. Russo M. Avizonis D. (2024). MTOR modulation induces selective perturbations in histone methylation which influence the anti-proliferative effects of mTOR inhibitors. iScience 27 (3), 109188. 10.1016/j.isci.2024.109188 38433910 PMC10904987

[B80] Kirkland J. L. Tchkonia T. (2020). Senolytic drugs: from discovery to translation. J. Intern. Med. 288 (5), 518–536. 10.1111/joim.13141 32686219 PMC7405395

[B81] Knezevic N. N. Candido K. D. Vlaeyen J. W. S. Van Zundert J. Cohen S. P. (2021). Low back pain. Lancet (London, England) 398 (10294), 78–92. 10.1016/S0140-6736(21)00733-9 34115979

[B82] Kong X. Gu H. Zhang Y. Meng Q. Li Q. Song K. (2025). β‐Mangostin attenuates TET2‐Mediated DNA demethylation of prkcg in the prevention of intervertebral disc degeneration. Adv. Sci. 12 (32), e05077. 10.1002/advs.202505077 PMC12407310 40558107

[B83] Kordowitzki P. (2025). Unveiling the relation between cellular ageing, epigenetics and cancer. Aging Dis. 17 (4), 1756–1763. 10.14336/ad.2025.0677 40586389 PMC13256693

[B84] Kovalev M. A. Mamaeva N. Y. Kristovskiy N. V. Feskin P. G. Vinnikov R. S. Oleinikov P. D. (2025). Epigenome engineering using dCas systems for biomedical applications and biotechnology: current achievements, opportunities and challenges. Int. J. Mol. Sci. 26 (13), 6371. 10.3390/ijms26136371 40650152 PMC12250444

[B85] Kumar M. A. Baba S. K. Sadida H. Q. Marzooqi S. A. Jerobin J. Altemani F. H. (2024). Extracellular vesicles as tools and targets in therapy for diseases. Signal Transduct. Target. Ther. 9 (1), 27. 10.1038/s41392-024-01735-1 38311623 PMC10838959

[B86] Kumari R. Jat P. (2021). Mechanisms of cellular senescence: cell cycle arrest and senescence associated secretory phenotype. Front. Cell Dev. Biol. 9, 645593. 10.3389/fcell.2021.645593 33855023 PMC8039141

[B87] Laberge R. M. Sun Y. Orjalo A. V. Patil C. K. Freund A. Zhou L. (2015). MTOR regulates the pro-tumorigenic senescence-associated secretory phenotype by promoting IL1A translation. Nat. Cell Biology 17 (8), 1049–1061. 10.1038/ncb3195 26147250 PMC4691706

[B88] Laplante M. Sabatini D. M. (2012). mTOR signaling in growth control and disease. Cell 149 (2), 274–293. 10.1016/j.cell.2012.03.017 22500797 PMC3331679

[B89] Lee E. Carreras-Gallo N. Lopez L. Turner L. Lin A. Mendez T. L. (2024). Exploring the effects of Dasatinib, Quercetin, and Fisetin on DNA methylation clocks: a longitudinal study on senolytic interventions. Aging 16 (4), 3088–3106. 10.18632/aging.205581 38393697 PMC10929829

[B90] Lei M. Lin H. Shi D. Hong P. Song H. Herman B. (2023). Molecular mechanism and therapeutic potential of HDAC9 in intervertebral disc degeneration. Cell. Mol. Biol. Lett. 28 (1), 104. 10.1186/s11658-023-00517-x 38093179 PMC10717711

[B91] Lérida Viso A. (2023). Pharmacological senolysis as a new therapeutic approach for the prevention of Doxorubicin-induced cardiotoxicity. RiuNet. 10.4995/thesis/10251/194709

[B92] Li G. Ma L. He S. Luo R. Wang B. Zhang W. (2022). WTAP-mediated m6A modification of lncRNA NORAD promotes intervertebral disc degeneration. Nat. Commun. 13 (1), 1469. 10.1038/s41467-022-28990-6 35304463 PMC8933458

[B93] Li Z. Ye D. Dai L. Xu Y. Wu H. Luo W. (2022). Single-cell RNA sequencing reveals the difference in human normal and degenerative nucleus pulposus tissue profiles and cellular interactions. Front. Cell Dev. Biol. 10, 910626. 10.3389/fcell.2022.910626 35874809 PMC9301035

[B94] Li Z. Yang H. Hai Y. Cheng Y. (2023). Regulatory effect of inflammatory mediators in intervertebral disc degeneration. Mediat. Inflamm. 2023, 1–19. 10.1155/2023/6210885 37101594 PMC10125773

[B95] Li T. Yang Y. Qi H. Cui W. Zhang L. Fu X. (2023). CRISPR/Cas9 therapeutics: progress and prospects. Signal Transduct. Target. Ther. 8, 1. 10.1038/s41392-023-01309-7 36646687 PMC9841506

[B96] Li L. Zhang G. Yang Z. Kang X. (2024). Stress-activated protein kinases in intervertebral disc degeneration: unraveling the impact of JNK and p38 MAPK. Biomolecules 14 (4), 393. 10.3390/biom14040393 38672411 PMC11047866

[B97] Li S. Du J. Huang Y. Gao S. Zhao Z. Chang Z. (2024). From hyperglycemia to intervertebral disc damage: exploring diabetic-induced disc degeneration. Front. Immunology 15, 1355503. 10.3389/fimmu.2024.1355503 PMC10912372 38444852

[B98] Li F. Li S. Shi Y. Lin F. Rui L. Shi J. (2024). Glutathione: a key regulator of extracellular matrix and cell death in intervertebral disc degeneration. Mediat. Inflammation 2024, 4482642. 10.1155/2024/4482642 PMC11473174 39403548

[B99] Li H. Kelley J. Ye Y. Ye Z. W. Townsend D. M. Zhang J. (2025). REDOX imbalance and oxidative stress in the intervertebral disc: the effect of mechanical stress and cigarette smoking on ER stress and mitochondrial dysfunction. Cells 14 (8), 613. 10.3390/cells14080613 40277939 PMC12025608

[B100] Liang C. Li H. Tao Y. Zhou X. Li F. Chen G. (2012). Responses of human adipose-derived mesenchymal stem cells to chemical microenvironment of the intervertebral disc. J. Transl. Med. 10, 49. 10.1186/1479-5876-10-49 22424131 PMC3338074

[B101] Liao G. Y. Lee M. T. Fan J. J. Hsiao P. W. Lee C. S. Su S. Y. (2019). Blockage of glutamine-dependent anaplerosis affects mTORC1/2 activity and ultimately leads to cellular senescence-like response. Biol. Open 8 (5), bio038257. 10.1242/bio.038257 31097446 PMC6550068

[B102] Liu C. Liu L. Yang M. Li B. Yi J. Ai X. (2020). A positive feedback loop between EZH2 and NOX4 regulates nucleus pulposus cell senescence in age-related intervertebral disc degeneration. Cell Div. 15, 2. 10.1186/s13008-020-0060-x 32025238 PMC6995653

[B103] Liu H. Hu J. Zheng Q. Feng X. Zhan F. Wang X. (2022). Piezo1 channels as force sensors in mechanical force-related chronic inflammation. Front. Immunol. 13, 816149. 10.3389/fimmu.2022.816149 35154133 PMC8826255

[B104] Liu R. Zhao E. Yu H. Yuan C. Abbas M. N. Cui H. (2023). Methylation across the central dogma in health and diseases: new therapeutic strategies. Signal Transduct. Target. Ther. 8 (1), 310. 10.1038/s41392-023-01528-y 37620312 PMC10449936

[B105] Liu S. Jia X. Hao J. Zhang D. Yang S. Dai B. (2023). Tissue engineering of JAK inhibitor‐loaded hierarchically biomimetic nanostructural scaffold targeting cellular senescence for aged bone defect repair and bone remolding. Adv. Healthc. Mater. 12 (30), e2301798. 10.1002/adhm.202301798 37667873

[B106] Liu J. Du C. Huang W. Lei Y. (2023). Injectable smart stimuli-responsive hydrogels: pioneering advancements in biomedical applications. Biomaterials Sci. 12 (1), 8–56. 10.1039/d3bm01352a 37969066

[B107] Liu Y. Dou Y. Sun X. Yang Q. (2024a). Mechanisms and therapeutic strategies for senescence-associated secretory phenotype in the intervertebral disc degeneration microenvironment. J. Orthop. Transl. 45, 56–65. 10.1016/j.jot.2024.02.003 PMC10943956 38495743

[B108] Liu Y. Wei C. Yang Y. Zhu Z. Ren Y. Pi R. (2024b). In situ chemical reprogramming of astrocytes into neurons: a new hope for the treatment of central neurodegenerative diseases? Eur. J. Pharmacol. 982, 176930. 10.1016/j.ejphar.2024.176930 39179093

[B109] Liu Y. Wang Y. Yan P. Cui N. Xu K. Liu D. (2025). NLRP3 inflammasome-mediated osteoarthritis: the role of epigenetics. Biology 14 (1), 71. 10.3390/biology14010071 39857301 PMC11761621

[B110] López K. G. Toledo Guzmán M. E. Sánchez E. O. García Carrancá A. (2019). mTORC1 as a regulator of mitochondrial functions and a therapeutic target in cancer. Front. Oncol. 9, 1373. 10.3389/fonc.2019.01373 31921637 PMC6923780

[B111] López-Otín C. Blasco M. A. Partridge L. Serrano M. Kroemer G. (2023). Hallmarks of aging: an expanding universe. Cell 186 (2), 243–278. 10.1016/j.cell.2022.11.001 36599349

[B112] Luo Z. Wei Z. Zhang G. Chen H. Li L. Kang X. (2023). Achilles’ Heel---The significance of maintaining microenvironmental homeostasis in the nucleus pulposus for intervertebral discs. Int. J. Mol. Sci. 24 (23), 16592. 10.3390/ijms242316592 38068915 PMC10706299

[B113] Lv F. J. Peng Y. Lim F. L. Sun Y. Lv M. Zhou L. (2016). Matrix metalloproteinase 12 is an indicator of intervertebral disc degeneration co-expressed with fibrotic markers. Osteoarthr. Cartilage 24 (10), 1826–1836. 10.1016/j.joca.2016.05.012 27211863

[B114] Ma Y. Xie H. Du X. Wang L. Jin X. Zhang Q. (2021). *In* vivo chemical reprogramming of astrocytes into neurons. Cell Discov. 7 (1), 12. 10.1038/s41421-021-00243-8 33649311 PMC7921425

[B115] Mahapatro A. Singh D. K. (2011). Biodegradable nanoparticles are excellent vehicle for site directed *in-vivo* delivery of drugs and vaccines. J. Nanobiotechnology 9 (1), 55. 10.1186/1477-3155-9-55 22123084 PMC3238292

[B116] Malekmohammadi S. Sedghi Aminabad N. Sabzi A. Zarebkohan A. Razavi M. Vosough M. (2021). Smart and biomimetic 3D and 4D printed composite hydrogels: opportunities for different biomedical applications. Biomedicines 9 (11), 1537. 10.3390/biomedicines9111537 34829766 PMC8615087

[B117] Maurer M. Lammerding J. (2019). The driving force: nuclear mechanotransduction in cellular function, fate, and disease. Annu. Rev. Biomed. Eng. 21 (1), 443–468. 10.1146/annurev-bioeng-060418-052139 30916994 PMC6815102

[B118] Meng Z. Tan J. Quyang X. Yin J. Yan Y. (2025). The relationship between biomechanical factors and intervertebral disc degeneration: a review. Am. J. Transl. Res. 17 (5), 3575–3585. 10.62347/DSJK1156 40535657 PMC12170379

[B119] Meng Y. Wu S. Liu S. Yang Y. (2025). Microenvironmental response-based treatment of osteoarthritis is a highly effective and durable program: a review. J. Orthop. Surg. Res. 20 (1), 966. 10.1186/s13018-025-06399-3 41194244 PMC12590603

[B120] Milazzo G. Mercatelli D. Di Muzio G. Triboli L. De Rosa P. Perini G. (2020). Histone deacetylases (HDACs): Evolution, specificity, role in transcriptional complexes, and pharmacological actionability. Genes 11 (5), 556. 10.3390/genes11050556 32429325 PMC7288346

[B121] Milheiro C. Moura M. L. Amendola M. Barbosa M. A. Caldeira J. (2025). Harnessing CRISPR potential for intervertebral disc regeneration strategies. Front. Bioeng. Biotechnol. 13, 1562412. 10.3389/fbioe.2025.1562412 40406584 PMC12095242

[B122] Mohanta T. K. Mishra A. K. Al-Harrasi A. (2021). The 3D genome: from structure to function. Int. J. Mol. Sci. 22 (21), 11585. 10.3390/ijms222111585 34769016 PMC8584255

[B123] Morein D. Erlichman N. Ben-Baruch A. (2020). Beyond cell motility: the expanding roles of chemokines and their receptors in malignancy. Front. Immunol. 11, 952. 10.3389/fimmu.2020.00952 32582148 PMC7287041

[B124] Nadeem J. Sultana R. Parveen A. Kim S. Y. (2025). Recent advances in anti‐aging therapeutic strategies targeting DNA damage response and senescence‐associated secretory phenotype‐linked signaling Cascade. Cell Biochem. Funct. 43 (3), e70046. 10.1002/cbf.70046 40008426

[B125] Nan X. Ng H. H. Johnson C. A. Laherty C. D. Turner B. M. Eisenman R. N. (1998). Transcriptional repression by the methyl-CpG-binding protein MeCP2 involves a histone deacetylase complex. Nature 393 (6683), 386–389. 10.1038/30764 9620804

[B126] National Center for Biotechnology Information (2026). PubChem patent summary for US-2005222684-A1, disc augmentation using materials that expand in situ. Available online at: https://pubchem.ncbi.nlm.nih.gov/patent/US-2005222684-A1 (Accessed May 11, 2026).

[B127] Nguyen H. T. (2023). Polymer-based hydrogels applied in drug delivery: an overview. Gels 9 (7), 523. 10.3390/gels9070523 37504402 PMC10379988

[B128] Ning Li Gao L. Zhang F. Li X. Wang T. (2021). Mechanical stretch induces annulus fibrosus cell senescence through activation of the RhoA/ROCK pathway. BioMed Res. Int. 2021, 5321121. 10.1155/2021/5321121 34840974 PMC8626192

[B129] Niu M. Ma F. Qian J. Li J. Wang T. Gao Y. (2018). N-cadherin attenuates nucleus pulposus cell senescence under high-magnitude compression. Mol. Med. Rep. 17 (2), 2879–2884. 10.3892/mmr.2017.8239 29257288 PMC5783503

[B130] Novais E. J. Tran V. A. Johnston S. N. Darris K. R. Roupas A. J. Sessions G. A. (2021). Long-term treatment with senolytic drugs Dasatinib and Quercetin ameliorates age-dependent intervertebral disc degeneration in mice. Nat. Commun. 12 (1), 5213. 10.1038/s41467-021-25453-2 34480023 PMC8417260

[B131] Núñez-Carro C. Blanco-Blanco M. Villagrán-Andrade K. M. Blanco F. J. de Andrés M. C. (2023). Epigenetics as a therapeutic target in osteoarthritis. Pharmaceuticals 16 (2), 156. 10.3390/ph16020156 37259307 PMC9964205

[B132] Paganella L. G. Bovone G. Cuni F. Labouesse C. Cui Y. Giampietro C. (2025). Injectable senolytic hydrogel depot for the clearance of senescent cells. Biomacromolecules 26 (2), 814–824. 10.1021/acs.biomac.4c00851 39783796 PMC11815846

[B133] Pandit P. Talbott J. F. Pedoia V. Dillon W. Majumdar S. (2016). T1ρ and T2 -based characterization of regional variations in intervertebral discs to detect early degenerative changes. J. Orthopaedic Res. 34 (8), 1373–1381. 10.1002/jor.23311 27227485 PMC6860013

[B134] Pattappa G. Li Z. Peroglio M. Wismer N. Alini M. Grad S. (2012). Diversity of intervertebral disc cells: phenotype and function. J. Anatomy 221 (6), 480–496. 10.1111/j.1469-7580.2012.01521.x 22686699 PMC3512276

[B135] Pirmoradi L. Shojaei S. Ghavami S. Zarepour A. Zarrabi A. (2023). Autophagy and biomaterials: a brief overview of the impact of autophagy in biomaterial applications. Pharmaceutics 15 (9), 2284. 10.3390/pharmaceutics15092284 37765253 PMC10536801

[B136] Pishavar E. Luo H. Naserifar M. Hashemi M. Toosi S. Atala A. (2021). Advanced hydrogels as exosome delivery systems for osteogenic differentiation of MSCs: application in bone regeneration. Int. J. Mol. Sci. 22 (12), 6203. 10.3390/ijms22126203 34201385 PMC8228022

[B137] Power H. T. Valtchev P. Dehghani F. Schindeler A. (2023). Strategies for senolytic drug discovery. Aging Cell 22 (10), e13948. 10.1111/acel.13948 37548098 PMC10577556

[B138] Rabinovitch M. (2023). Are senolytic agents guilty of overkill or inappropriate age discrimination? Circulation 147 (8), 667–668. 10.1161/circulationaha.122.060247 36802881 PMC10027375

[B139] Ren Q. Chen L. Ma Y. Huang Y. Wang S. (2025). Immune microenvironment in intervertebral disc degeneration: pathophysiology and therapeutic potential. Front. Immunology 16 (4 Jul), 1563635. 10.3389/fimmu.2025.1563635 PMC12271889 40688090

[B140] Resseguie E. A. Staversky R. J. Brookes P. S. O'Reilly M. A. (2015). Hyperoxia activates ATM independent from mitochondrial ROS and dysfunction. Redox Biol. 5, 176–185. 10.1016/j.redox.2015.04.012 25967673 PMC4430709

[B141] Rider S. M. Mizuno S. Kang J. D. (2018). Molecular mechanisms of intervertebral disc degeneration. Spine Surgery Related Research 3 (1), 1–11. 10.22603/ssrr.2017-0095 31435545 PMC6690117

[B142] Risbud M. V. Shapiro I. M. (2014). Role of cytokines in intervertebral disc degeneration: pain and disc content. Nat. Rev. Rheumatol. 10 (1), 44–56. 10.1038/nrrheum.2013.160 24166242 PMC4151534

[B143] Risbud M. V. Schaer T. P. Shapiro I. M. (2010). Toward an understanding of the role of notochordal cells in the adult intervertebral disc: from discord to accord. Dev. Dyn. 239 (8), 2141–2148. 10.1002/dvdy.22350 20568241 PMC3634351

[B144] Rohanifar M. Clayton S. W. Easson G. W. Patil D. S. Lee F. Jing L. (2022). Single cell RNA-sequence analyses reveal uniquely expressed genes and heterogeneous immune cell involvement in the rat model of intervertebral disc degeneration. Appl. Sci. 12 (16), 8244. 10.3390/app12168244 36451894 PMC9706593

[B145] Samanta A. Lufkin T. Kraus P. (2023). Intervertebral disc degeneration---Current therapeutic options and challenges. Front. Public Health 11, 1156749. 10.3389/fpubh.2023.1156749 37483952 PMC10359191

[B146] Sang Y. Zhao H. Wu J. Zhang T. Xu W. Liu K. (2025). Downregulation of aquaporin 3 promotes hyperosmolarity-induced apoptosis of nucleus pulposus cells through PI3K/Akt/mTOR pathway suppression. Front. Genet. 16, 1665899. 10.3389/fgene.2025.1665899 41262548 PMC12626380

[B147] Saul D. Kosinsky R. L. (2021). Epigenetics of aging and aging-associated diseases. Int. J. Mol. Sci. 22 (1), 401. 10.3390/ijms22010401 33401659 PMC7794926

[B148] Scalise M. Marino F. Salerno L. Cianflone E. Molinaro C. Salerno N. (2021). From spheroids to organoids: the next generation of model systems of human cardiac regeneration in a dish. Int. J. Mol. Sci. 22 (24), 13180. 10.3390/ijms222413180 34947977 PMC8708686

[B149] Seki S. Iwasaki M. Makino H. Yahara Y. Miyazaki Y. Kamei K. (2022). Direct reprogramming and induction of human dermal fibroblasts to differentiate into iPS-Derived nucleus pulposus-like cells in 3D culture. Int. J. Mol. Sci. 23 (7), 4059. 10.3390/ijms23074059 35409417 PMC8999916

[B150] Sgro A. Blancafort P. (2020). Epigenome engineering: new technologies for precision medicine. Nucleic Acids Res. 48 (22), 12453–12482. 10.1093/nar/gkaa1000 33196851 PMC7736826

[B151] Shao J. Yu M. Jiang L. Wu F. Liu X. (2016). Sequencing and bioinformatics analysis of the differentially expressed genes in herniated discs with or without calcification. Int. J. Mol. Med. 39 (1), 81–90. 10.3892/ijmm.2016.2821 27959380 PMC5179188

[B152] Shao Z. Ni L. Hu S. Xu T. Meftah Z. Yu Z. (2021). RNA-binding protein HuR suppresses senescence through Atg7 mediated autophagy activation in diabetic intervertebral disc degeneration. Cell Proliferation 54 (2), e12975. 10.1111/cpr.12975 33372336 PMC7848958

[B153] Shetty A. P. Ramachandran K. Ponnuraj S. (2026). Genomics in spine surgery. Indian Spine J. 9 (1), 48–54. 10.4103/isj.isj_86_25

[B154] Sheyn D. Ben-David S. Tawackoli W. Zhou Z. Salehi K. Bez M. (2019). Human iPSCs can be differentiated into notochordal cells that reduce intervertebral disc degeneration in a porcine model. Theranostics 9 (25), 7506–7524. 10.7150/thno.34898 31695783 PMC6831475

[B155] Shi S. Kang X. J. Zhou Z. He Z. M. Zheng S. He S. S. (2022). Excessive mechanical stress-induced intervertebral disc degeneration is related to Piezo1 overexpression triggering the imbalance of autophagy/apoptosis in human nucleus pulpous. Arthritis Res. Ther. 24 (1), 119. 10.1186/s13075-022-02804-y 35606793 PMC9125856

[B156] Shi X. Li P. Wu X. Shu J. (2023). Whole-transcriptome sequencing identifies key differentially expressed circRNAs/lncRNAs/miRNAs/mRNAs and linked ceRNA networks in adult degenerative scoliosis. Front. Mol. Neurosci. 16, 1038816. 10.3389/fnmol.2023.1038816 37063366 PMC10098162

[B157] Shukla S. Tekwani B. L. (2020). Histone deacetylases inhibitors in neurodegenerative diseases, neuroprotection and neuronal differentiation. Front. Pharmacol. 11, 537. 10.3389/fphar.2020.00537 32390854 PMC7194116

[B158] Silwal P. Nguyen-Thai A. M. Mohammad H. A. Wang Y. Robbins P. D. Lee J. Y. (2023). Cellular senescence in intervertebral disc aging and degeneration: molecular mechanisms and potential therapeutic opportunities. Biomolecules 13 (4), 686. 10.3390/biom13040686 37189433 PMC10135543

[B159] Simonson B. Das S. (2015). MicroRNA therapeutics: the next magic bullet? Mini-Rev. Med. Chem. 15 (6), 467–474. 10.2174/1389557515666150324123208 25807941 PMC4410078

[B160] Skubutyte R. Markova D. Freeman T. A. Anderson D. G. Dion A. S. Williams C. J. (2010). Hypoxia-inducible factor regulation of ANK expression in nucleus pulposus cells: possible implications in controlling dystrophic mineralization in the intervertebral disc. Arthritis Rheumatism 62 (9), 2707–2715. 10.1002/art.27558 20496369 PMC3065237

[B161] Son S. M. Park S. J. Breusegem S. Y. Larrieu D. Rubinsztein D. C. (2024). p300 nucleocytoplasmic shuttling underlies mTORC1 hyperactivation in Hutchinson-Gilford progeria syndrome. Nat. Cell Biology 26 (2), 235–249. 10.1038/s41556-023-01338-y 38267537 PMC10866696

[B162] Song J. Chen Z. H. Zheng C. J. Song K. H. Xu G. Y. Xu S. (2020). Exosome-Transported circRNA_0000253 competitively adsorbs MicroRNA-141-5p and increases IDD. Mol. Ther. Nucleic Acids 21, 1087–1099. 10.1016/j.omtn.2020.07.039 32858458 PMC7473879

[B163] Soto-Palma C. Niedernhofer L. J. Faulk C. D. Dong X. (2022). Epigenetics, DNA damage, and aging. J. Clin. Invest. 132, 16. 10.1172/jci158446 35968782 PMC9374376

[B164] Steinle H. Behring A. Schlensak C. Wendel H. P. Avci-Adali M. (2016). Concise review: application of In Vitro transcribed messenger RNA for cellular engineering and reprogramming: progress and challenges. Stem Cells 35 (1), 68–79. 10.1002/stem.2402 27250673

[B165] Stirnimann A. Schlagenhof L. Gantenbein B. Ille F. (2025). Advancing intervertebral disc biology via omics: implications for nucleus pulposus progenitor cell‐based regeneration. JOR Spine 8 (4), e70130. 10.1002/jsp2.70130 41112064 PMC12530025

[B166] Stojanović B. Jovanovic I. Dimitrijevic Stojanovic M. Stojanovic B. S. Kovacevic V. Radosavljevic I. (2025). Oxidative stress-driven cellular senescence: mechanistic crosstalk and therapeutic Horizons. Antioxidants 14 (8), 987. 10.3390/antiox14080987 40867884 PMC12383077

[B167] Sun J. Gui Y. Zhou S. Zheng X. L. (2023). Unlocking the secrets of aging: epigenetic reader BRD4 as the target to combatting aging-related diseases. J. Adv. Res. 63, 207–218. 10.1016/j.jare.2023.11.006 37956861 PMC11379999

[B168] Sun M. Clayton N. Alam S. Asmussen N. Wong A. Kim J. H. (2023). Selective BET inhibitor RVX‐208 ameliorates periodontal inflammation and bone loss. J. Clin. Periodontology 50 (12), 1658–1669. 10.1111/jcpe.13887 37855275

[B169] Swahn H. Mertens J. Olmer M. Myers K. Mondala T. S. Natarajan P. (2024). Shared and compartment-specific processes in nucleus pulposus and annulus fibrosus during intervertebral disc degeneration. Adv. Sci. 11 (17), e2309032. 10.1002/advs.202309032 PMC11077672 38403470

[B170] Tessarz P. Kouzarides T. (2014). Histone core modifications regulating nucleosome structure and dynamics. Nat. Rev. Mol. Cell Biol. 15 (11), 703–708. 10.1038/nrm3890 25315270

[B171] Tessier S. Risbud M. V. (2020). Understanding embryonic development for cell‐based therapies of intervertebral disc degeneration: toward an effort to treat disc degeneration subphenotypes. Dev. Dyn. 250 (3), 302–317. 10.1002/dvdy.217 32564440

[B172] Tilotta V. Vadalà G. Ambrosio L. Cicione C. Di Giacomo G. Russo F. (2023). Mesenchymal stem cell-derived secretome enhances nucleus pulposus cell metabolism and modulates extracellular matrix gene expression in vitro. Front. Bioeng. Biotechnol. 11, 1152207. 10.3389/fbioe.2023.1152207 37008028 PMC10060656

[B173] Tong L. Yu H. Huang X. Shen J. Xiao G. Chen L. (2022). Current understanding of osteoarthritis pathogenesis and relevant new approaches. Bone Res. 10 (1), 60. 10.1038/s41413-022-00226-9 36127328 PMC9489702

[B174] Tsai M. C. Manor O. Wan Y. Mosammaparast N. Wang J. K. Lan F. (2010). Long noncoding RNA as modular scaffold of histone modification complexes. Science 329 (5992), 689–693. 10.1126/science.1192002 20616235 PMC2967777

[B175] Tsujimoto R. Yurube T. Takeoka Y. Kanda Y. Miyazaki K. Ohnishi H. (2022). Involvement of autophagy in the maintenance of rat intervertebral disc homeostasis: an in-vitro and in-vivo RNA interference study of Atg5. Osteoarthr. Cartilage 30 (3), 481–493. 10.1016/j.joca.2021.12.004 34958937

[B176] Tu Ji Li W. Yang S. Yang P. Yan Q. Wang S. (2021). Single‐cell transcriptome profiling reveals multicellular ecosystem of nucleus pulposus during degeneration progression. Adv. Sci. 9 (3), e2103631. 10.1002/advs.202103631 PMC8787427 34825784

[B177] Tylutka A. Walas Ł. Zembron-Lacny A. (2024). Level of IL-6, TNF, and IL-1β and age-related diseases: a systematic review and meta-analysis. Front. Immunol. 15, 1330386. 10.3389/fimmu.2024.1330386 38495887 PMC10943692

[B178] Vadalà G. Ambrosio L. Russo F. Papalia R. Denaro V. (2019). Interaction between mesenchymal stem cells and intervertebral disc microenvironment: from cell therapy to tissue engineering. Stem Cells Int. 2019, 2376172. 10.1155/2019/2376172 32587618 PMC7294366

[B179] Vedova L. D. Baron G. Morazzoni P. Aldini G. Gado F. (2025). The potential of polyphenols in modulating the cellular senescence process: implications and mechanism of action. Pharmaceuticals 18 (2), 138. 10.3390/ph18020138 40005954 PMC11858549

[B180] Veronesi F. Salamanna F. Tedesco G. Ruffilli A. Rosa F. Faldini C. (2026). Mapping the degenerating intervertebral disc: a systematic review of histological evidence. Front. Med. 13, 1753988. 10.3389/fmed.2026.1753988 PMC12977079 41822885

[B181] Vo N. V. Hartman R. A. Patil P. R. Risbud M. V. Kletsas D. Iatridis J. C. (2016). Molecular mechanisms of biological aging in intervertebral discs. J. Orthopaedic Res. 34 (8), 1289–1306. 10.1002/jor.23195 26890203 PMC4988945

[B182] Voigt P. Tee W. W. Reinberg D. (2013). A double take on bivalent promoters. Genes Dev. 27 (12), 1318–1338. 10.1101/gad.219626.113 23788621 PMC3701188

[B183] Wan W. You Z. Xu Y. Zhou L. Guan Z. Peng C. (2017). mTORC1 phosphorylates acetyltransferase p300 to regulate autophagy and lipogenesis. Mol. Cell 68 (2), 323–335.e6. 10.1016/j.molcel.2017.09.020 29033323

[B184] Wang H. La Russa M. Qi L. S. (2016). CRISPR/Cas9 in genome editing and beyond. Annu. Rev. Biochem. 85 (1), 227–264. 10.1146/annurev-biochem-060815-014607 27145843 PMC13384723

[B185] Wang M. Jiang S. Wu W. Yu F. Chang W. Li P. (2018). Non-coding RNAs function as immune regulators in teleost fish. Front. Immunol. 9, 2801. 10.3389/fimmu.2018.02801 30546368 PMC6279911

[B186] Wang W. Wang Y. Deng G. Ma J. Huang X. Yu J. (2018). Transplantation of hypoxic-preconditioned bone mesenchymal stem cells retards intervertebral disc degeneration via enhancing implanted cell survival and migration in rats. Stem Cells Int. 2018, 7564159. 10.1155/2018/7564159 29535780 PMC5832130

[B187] Wang L. Cho K. B. Li Y. Tao G. Xie Z. Guo B. (2019). Long noncoding RNA (lncRNA)-Mediated competing endogenous RNA networks provide novel potential biomarkers and therapeutic targets for colorectal cancer. Int. J. Mol. Sci. 20 (22), 5758. 10.3390/ijms20225758 31744051 PMC6888455

[B188] Wang N. Wu R. Tang D. Kang R. (2021). The BET family in immunity and disease. Signal Transduct. Target. Ther. 6 (1), 23. 10.1038/s41392-020-00384-4 33462181 PMC7813845

[B189] Wang J. Huang Y. Huang L. Shi K. Wang J. Zhu C. (2021). Novel biomarkers of intervertebral disc cells and evidence of stem cells in the intervertebral disc. Osteoarthr. Cartil. 29 (3), 389–401. 10.1016/j.joca.2020.12.005 33338640

[B190] Wang Y. Zhang Y. Chen K. Shao F. Wu Y. Guo C. (2021). Injectable nanostructured colloidal gels resembling native nucleus pulposus as carriers of mesenchymal stem cells for the repair of degenerated intervertebral discs. Materials science and engineering. C, Mater. Biological Appl. 128, 112343. 10.1016/j.msec.2021.112343 34474893

[B191] Wang C. Cui L. Gu Q. Guo S. Zhu B. Liu X. (2022). The mechanism and function of miRNA in intervertebral disc degeneration. Orthop. Surg. 14 (3), 463–471. 10.1111/os.13204 35142050 PMC8926997

[B192] Wang D. Wu W. Callen E. Pavani R. Zolnerowich N. Kodali S. (2022). Active DNA demethylation promotes cell fate specification and the DNA damage response. Science 378 (6623), 983–989. 10.1126/science.add9838 36454826 PMC10196940

[B193] Wang Z. Zhang Z. C. Wu Y. Y. Pi Y. N. Lou S. H. Liu T. B. (2023). Bromodomain and extraterminal (BET) proteins: biological functions, diseases and targeted therapy. Signal Transduct. Target. Ther. 8 (1), 420. 10.1038/s41392-023-01647-6 37926722 PMC10625992

[B194] Wang W. Liu L. Ma W. Zhao L. Huang L. Zhou D. (2024). An anti-senescence hydrogel with pH-responsive drug release for mitigating intervertebral disc degeneration and low back pain. Bioact. Materials 41, 355–370. 10.1016/j.bioactmat.2024.07.031 PMC11338064 39171275

[B195] Wang H. Cheng P. Wang J. Lv H. Han J. Hou Z. (2025). Advances in spatial transcriptomics and its application in the musculoskeletal system. Bone Res. 13 (1), 54. 10.1038/s41413-025-00429-w 40379648 PMC12084619

[B196] Wang Z. L. Naruse K. Takahashi K. (2026). Bridging the gap between static histology and dynamic Organ-on-a-Chip models. Pathophysiology 33 (1), 10. 10.3390/pathophysiology33010010 41718388 PMC12922035

[B197] Warren L. Ni Y. Wang J. Guo X. (2012). Feeder-free derivation of human induced pluripotent stem cells with messenger RNA. Sci. Rep. 2 (1), 657. 10.1038/srep00657 22984641 PMC3442198

[B198] Weber K. T. Alipui D. O. Sison C. P. Bloom O. Quraishi S. Overby M. C. (2016). Serum levels of the proinflammatory cytokine interleukin-6 vary based on diagnoses in individuals with lumbar intervertebral disc diseases. Arthritis Res. Ther. 18, 3. 10.1186/s13075-015-0887-8 26743937 PMC4718017

[B199] Wijnen A. J. van. (2019). Epigenetics as a new frontier in orthopedic regenerative medicine and oncology. J. Orthop. Res. 37 (7), 1465–1474. 10.1002/jor.24305 30977555 PMC6588446

[B200] Winkler J. Raina K. Altieri M. Dong H. Wang J. Chen X. (2016). PROTAC BET degraders are more broadly effective than BET inhibitors. Eur. J. Cancer 69, S10. 10.1016/s0959-8049(16)32621-1

[B201] Wong R. C. B. (2017). Neuroregeneration using in vivo cellular reprogramming. Neural Regen. Res. 12 (7), 1073. 10.4103/1673-5374.211182 28852386 PMC5558483

[B202] Wu Y. Shen S. Shi Y. Tian N. Zhou Y. Zhang X. (2022). Senolytics: eliminating senescent cells and alleviating intervertebral disc degeneration. Front. Bioeng. Biotechnol. 10, 823945. 10.3389/fbioe.2022.823945 35309994 PMC8924288

[B203] Xia K. Gong Z. Zhu J. Yu W. Wang Y. Wang J. (2019). Differentiation of pluripotent stem cells into nucleus pulposus progenitor cells for intervertebral disc regeneration. Curr. Stem Cell Res. Ther. 14 (1), 57–64. 10.2174/1574888X13666180918095121 30227822

[B204] Xiang Q. Wang J. Cheng Z. Zhao K. Gan W. Chen Y. (2023). Hsa_circ_0001946 ameliorates mechanical stress-induced intervertebral disk degeneration Via targeting miR-432-5p and SOX9. Spine 48 (23), E401–E408. 10.1097/BRS.0000000000004777 37555796 PMC10624407

[B205] Xiang H. Yan C. Lei Y. Lai N. Zhang P. Jiang K. (2025). Aging‐regulating microspheres for enhancing mitochondrial biogenesis in early senescence and clearing late senescent cells. Adv. Funct. Mater. 35, 51. 10.1002/adfm.202506677

[B206] Xiang Q. Zhan J. Tian S. Zhao Y. Wu Z. Lin J. (2025). Human iPSCs derived MSCs-Secreted exosomes modulate senescent nucleus pulposus cells induced macrophage polarization via metabolic reprogramming to mitigate intervertebral disc degeneration. Adv. Sci. 12 (36), e04347. 10.1002/advs.202504347 PMC12463037 40619582

[B207] Xiao Q. Nobre A. Piñeiro P. Berciano-Guerrero M. Á. Alba E. Cobo M. (2020). Genetic and epigenetic biomarkers of immune checkpoint blockade response. J. Clin. Med. 9 (1), 286. 10.3390/jcm9010286 31968651 PMC7019273

[B208] Xie Y. Guan Q. Guo J. Chen Y. Yin Y. Han X. (2022). Hydrogels for exosome delivery in biomedical applications. Gels 8 (6), 328. 10.3390/gels8060328 35735672 PMC9223116

[B209] Xu Y.-Di Liang X. C. Li Z. P. Wu Z. S. Yang J. Jia S. Z. (2024). Apoptotic body-inspired nanotherapeutics efficiently attenuate osteoarthritis by targeting BRD4-regulated synovial macrophage polarization. Biomaterials 306, 122483. 10.1016/j.biomaterials.2024.122483 38330742

[B210] Yang C. Wu D. Gao L. Liu X. Jin Y. Wang D. (2016). Competing endogenous RNA networks in human cancer: hypothesis, validation, and perspectives. Oncotarget 7 (12), 13479–13490. 10.18632/oncotarget.7266 26872371 PMC4924655

[B211] Yang J. E. Zhao K. H. Qu Y. Zou Y. C. (2022). Increased serum CXCL10 levels are associated with clinical severity and radiographic progression in patients with lumbar disc degeneration. Clin. Chimica Acta; International Journal Clinical Chemistry 525, 15–22. 10.1016/j.cca.2021.12.006 34902344

[B212] Yang S. Hu H. Kung H. Zou R. Dai Y. Hu Y. (2023). Organoids: the current status and biomedical applications. MedComm 4.3, e274. 10.1002/mco2.274 37215622 PMC10192887

[B213] Yang H. Chen X. Chen J. Dong Y. Huang Y. Qin L. (2024). The pathogenesis and targeted therapies of intervertebral disc degeneration induced by cartilage endplate inflammation. Front. Cell Developmental Biology 12 (2 Dec), 1492870. 10.3389/fcell.2024.1492870 PMC11647014 39687521

[B214] Yang Y. Li H. Zuo J. Lei F. (2025). Mechanistic interactions driving nucleus pulposus cell senescence in intervertebral disc degeneration: a multi‐axial perspective of mechanical, immune, and metabolic pathways. JOR Spine 8 (3), e70089. 10.1002/jsp2.70089 40606198 PMC12216508

[B215] Yao Q. Lei Y. Zhang Y. Chen H. Dong X. Ye Z. (2024). EZH2-H3K27me3-Mediated epigenetic silencing of DKK1 induces nucleus pulposus cell pyroptosis in intervertebral disc degeneration by activating NLRP3 and NAIP/NLRC4. Inflammation 48 (2), 902–918. 10.1007/s10753-024-02096-1 39052181

[B216] Ye G. Xie T. Zeng H. Wang P. Li J. Zheng G. (2020). Oxidative stress-mediated mitochondrial dysfunction facilitates mesenchymal stem cell senescence in ankylosing spondylitis. Cell Death Dis. 11 (9), 775. 10.1038/s41419-020-02993-x 32943613 PMC7498590

[B217] Yetisgin A. A. Cetinel S. Zuvin M. Kosar A. Kutlu O. (2020). Therapeutic nanoparticles and their targeted delivery applications. Molecules 25 (9), 2193. 10.3390/molecules25092193 32397080 PMC7248934

[B218] Jin Y. Liu Z. Li Z. Li H. Zhu C. Li R. (2022). Histone demethylase JMJD3 downregulation protects against aberrant force-induced osteoarthritis through epigenetic control of NR4A1. Int. J. Oral Sci. 14 (1), 34. 10.1038/s41368-022-00190-4 35831280 PMC9279410

[B219] Yurube T. Buchser W. J. Zhang Z. Silwal P. Lotze M. T. Kang J. D. (2024). Rapamycin mitigates inflammation-mediated disc matrix homeostatic imbalance by inhibiting mTORC1 and inducing autophagy through Akt activation. JOR Spine 7 (1), e1303. 10.1002/jsp2.1303 38222800 PMC10782056

[B220] Zhu Y. Liang Y. Zhu H. Lian C. Wang L. Wang Y. (2017). The generation and functional characterization of induced pluripotent stem cells from human intervertebral disc nucleus pulposus cells. Oncotarget 8 (26), 42700–42711. 10.18632/oncotarget.17446 28498811 PMC5522099

[B221] Zhang W. Hu J. Wu H. Lin X. Cai L. (2025). Stimuli-responsive hydrogel dressing for wound healing. Apl. Mater. 13 (1), 010601. 10.1063/5.0245545

[B222] Zhan S. Wang K. Song Y. Li S. Yin H. Luo R. (2019). Long non-coding RNA HOTAIR modulates intervertebral disc degenerative changes via Wnt/β-catenin pathway. Arthritis Research and Therapy 21 (1), 201. 10.1186/s13075-019-1986-8 31481088 PMC6724301

[B223] Zhang T. Cooper S. Brockdorff N. (2015). The interplay of histone modifications -- writers that read. EMBO Rep. 16 (11), 1467–1481. 10.15252/embr.201540945 26474904 PMC4641500

[B224] Zhang X. Lu H. Hong W. Liu L. Wang S. Zhou M. (2018). Tyrphostin B42 attenuates trichostatin A-mediated resistance in pancreatic cancer cells by antagonizing IL-6/JAK2/STAT3 signaling. Oncol. Rep. 39 (4), 1892–1900. 10.3892/or.2018.6241 29393444

[B225] Zhang X. Wang W. Zhu W. Dong J. Cheng Y. Yin Z. (2019). Mechanisms and functions of long non-coding RNAs at multiple regulatory levels. Int. J. Mol. Sci. 20 (22), 5573. 10.3390/ijms20225573 31717266 PMC6888083

[B226] Zhang Y. Yang B. Wang J. Cheng F. Shi K. Ying L. (2020). Cell senescence: a nonnegligible cell state under survival stress in pathology of intervertebral disc degeneration. Oxidative Med. Cell. Longev. 2020, 1–12. 10.1155/2020/9503562 32934764 PMC7479476

[B227] Zhang J. Zhang W. Sun T. Wang J. Li Y. Liu J. (2022). The influence of intervertebral disc microenvironment on the biological behavior of engrafted mesenchymal stem cells. Stem Cells Int. 2022, 1–24. 10.1155/2022/8671482 PMC9663214 36387746

[B228] Zhang L. Pitcher L. E. Yousefzadeh M. J. Niedernhofer L. J. Robbins P. D. Zhu Y. (2022). Cellular senescence: a key therapeutic target in aging and diseases. J. Clin. Investigation 132, 15. 10.1172/jci158450 PMC9337830 35912854

[B229] Zhang S. Song S. Cui W. Liu X. Sun Z. (2022). Mechanism of long noncoding RNA HOTAIR in nucleus pulposus cell autophagy and apoptosis in intervertebral disc degeneration. Evidence-based Complementary Alternative Med. 2022, 8504601–8504618. 10.1155/2022/8504601 PMC8752263 35027936

[B230] Zhang J. Wang S. Liu B. (2023). New insights into the genetics and epigenetics of aging plasticity. Genes 14 (2), 329. 10.3390/genes14020329 36833255 PMC9956228

[B231] Zhang X. Zhang Z. Zou X. Wang Y. Qi J. Han S. (2024). Unraveling the mechanisms of intervertebral disc degeneration: an exploration of the p38 MAPK signaling pathway. Front. Cell Dev. Biol. 11, 1324561. 10.3389/fcell.2023.1324561 38313000 PMC10834758

[B232] Zhang S.-P. Tong M. Li S. Zhang B. Zhang W. Wang R. (2024). The role of microvascular variations in the process of intervertebral disk degeneration and its regulatory mechanisms: a literature review. Orthop. Surg. 16 (11), 2587–2597. 10.1111/os.14209 39205477 PMC11541140

[B233] Zhang F. Guo J. Yu S. Zheng Y. Duan M. Zhao L. (2024). Cellular senescence and metabolic reprogramming: unraveling the intricate crosstalk in the immunosuppressive tumor microenvironment. Cancer Commun. 44 (9), 929–966. 10.1002/cac2.12591 PMC11492308 38997794

[B234] Zhang G. Li L. Yang Z. Cao Z. Hu X. Wang Y. (2025). BRD4/MAP2K7/PGF signaling axis promotes senescence and extracellular matrix metabolism of nucleus pulposus cells in intervertebral disk degeneration. Aging Cell 24 (6), e70034. 10.1111/acel.70034 40130513 PMC12151915

[B235] Zhang J. Zou J. Ren J. (2025). Recent advances in glycopeptide hydrogels: design, biological functions, and biomedical applications. Front. Bioeng. Biotechnol. 13, 1577192. 10.3389/fbioe.2025.1577192 40625852 PMC12230054

[B236] Zhong W. Xu L. He N. Liao W. Liu C. Xu K. (2026). A ROS and pH dual - responsive hydrogel-nanoparticle system restores intervertebral disc and alleviates related pain by modulating senescence and panoptosis of nucleus pulposus cells. Mater. Today. Bio. 37, 102907. 10.1016/j.mtbio.2026.102907 41716350 PMC12914207

[B237] Zhu Yi Tchkonia T. Pirtskhalava T. Gower A. C. Ding H. Giorgadze N. (2015). The Achilles’ heel of senescent cells: from transcriptome to senolytic drugs. Aging Cell 14 (4), 644–658. 10.1111/acel.12344 25754370 PMC4531078

[B238] Zhu X. Chen Z. Shen W. Huang G. Sedivy J. M. Wang H. (2021). Inflammation, epigenetics, and metabolism converge to cell senescence and ageing: the regulation and intervention. Signal Transduct. Target. Ther. 6 (1), 245. 10.1038/s41392-021-00646-9 34176928 PMC8236488

[B239] Zhu D. Liang H. Du Z. Liu Q. Li G. Zhang W. (2024). Altered metabolism and inflammation driven by post-translational modifications in intervertebral disc degeneration. Research 7, 0350. 10.34133/research.0350 38585329 PMC10997488

[B240] Zhu D. Liang H. Tong B. Du Z. Li G. Zhang W. (2025). DDX1 methylation mediated MATR3 splicing regulates intervertebral disc degeneration by initiating chromatin reprogramming. Nat. Commun. 16 (1), 6153. 10.1038/s41467-025-61486-7 40610464 PMC12229552

